# Targeting Cancer-Associated Fibroblasts in Prostate Cancer: Recent Advances and Therapeutic Opportunities

**DOI:** 10.3390/cancers18010151

**Published:** 2025-12-31

**Authors:** Peng Chen, Junhao Chen, Peiqin Zhan, Xinni Ye, Li Zhao, Zhongsong Zhang, Jieming Zuo, Hongjin Shi, Xiangyun Li, Songhong Wu, Yuanzhi Fu, Haifeng Wang, Shi Fu

**Affiliations:** 1 Department of Urology, The Second Affiliated Hospital of Kunming Medical University, Kunming 650106, China; 469374619@qq.com (P.C.); 20231042@kmmu.edu.cn (J.C.); 20231037@kmmu.edu.cn (P.Z.); 20241098@kmmu.edu.cn (X.Y.); 20231951@kmmu.edu.cn (J.Z.); 20190797@kmmu.edu.cn (H.S.); 20220985@kmmu.edu.cn (X.L.); wsh5346@163.com (S.W.); mackgod999@gmail.com (Y.F.); 2 Department of Anesthesiology, The First Affiliated Hospital of Kunming Medical University, Kunming 650032, China; zhaoli3@kmmu.edu.cn; 3 School of Clinical Medicine, Chengdu Medical College, Chengdu 610550, China; zzscmc1122@163.com; 4 Department of Clinical Medicine, Kunming University of Science and Technology, Kunming 650500, China

**Keywords:** prostate cancer, cancer-associated fibroblasts, tumor microenvironment, stromal heterogeneity, castration-resistant prostate cancer, therapeutic resistance, targeted therapy

## Abstract

Advanced prostate cancer, particularly castration-resistant disease, remains challenging to treat due to intratumoral heterogeneity, immune exclusion, and a suppressive tumor microenvironment. Within this ecosystem, cancer-associated fibroblasts shape tumor–stroma communication, but their marked heterogeneity and plasticity complicate classification and make indiscriminate fibroblast depletion potentially ineffective or even harmful. This review summarizes recent progress in fibroblast origins, functional subtypes, and fibroblast-driven mechanisms that promote tumor progression and therapy resistance, as well as emerging therapeutic opportunities in prostate cancer. We conducted a structured literature search of PubMed, ScienceDirect, and major publisher platforms (including Nature and SpringerLink) from database inception to 15 February 2025, supplemented by targeted manual screening of reference lists. Evidence from single-cell/spatial-omics and mechanistic studies indicates that prostate tumors contain multiple fibroblast programs that occupy distinct niches yet can interconvert. Across these studies, it was found that these fibroblasts contribute to immune suppression, extracellular matrix remodeling and stromal barrier formation, angiogenesis, and metabolic support, collectively limiting drug penetration and reinforcing immune evasion; therapeutic pressure can further rewire fibroblast states and resistance-associated signaling. Overall, the literature supports a shift toward function- and subtype-directed intervention rather than “one-size-fits-all” targeting, with promising directions including precision targeting and reversible reprogramming, rational combination strategies, and localized delivery approaches that reduce stromal barriers while preserving tissue homeostasis in high-risk and treatment-refractory prostate cancer.

## Simple Summary

Prostate cancer can become difficult to treat when it adapts to therapy and spreads, leading to treatment-resistant disease. A key reason for this is that cancer cells do not act alone: they are supported by nearby “helper” cells that shape the tumor environment. Among these, cancer-associated fibroblasts can build a dense scaffold around tumors, release signals that fuel cancer growth, and weaken immune responses, which may reduce the effectiveness of medicines. This review aims to explain what these fibroblasts are, how they change during prostate cancer progression, and why different fibroblast types may require different treatment approaches. By summarizing recent biological insights and emerging therapeutic strategies, we highlight how targeting the tumor-supporting environment—rather than cancer cells alone—could help design more effective combination treatments and guide future research toward more personalized care.

## 1. Introduction

Prostate cancer (PCa) is the second most commonly diagnosed malignancy and the fifth leading cause of cancer-related death among men worldwide. In 2022, approximately 1.5 million new cases and 397,000 deaths were reported globally, and demographic projections suggest that the annual burden may rise substantially by 2040 [1,2]. These trends underscore the need for early, system-level planning to strengthen PCa screening, diagnostic pathways, and treatment capacity [3].

Despite improvements in prostate-specific antigen (PSA) testing, multiparametric magnetic resonance imaging (MRI), and risk-adapted local therapies, a substantial fraction of patients still progress to locally advanced or metastatic disease [4], survival gains for advanced disease—particularly castration-resistant prostate cancer (CRPC)—remain limited [5,6,7]. Recent systematic studies show that cancer-associated fibroblasts (CAFs), key components of the PCa stromal ecosystem, promote tumor progression via paracrine signaling, ECM remodeling, and altered tissue biomechanics. Moreover, under the selective pressure of endocrine therapy, CAFs states can be reprogrammed [8,9,10]. Collectively, these observations suggest that delineating the functional heterogeneity of CAFs in PCa, and the dynamics of their interactions with tumor cells and the microenvironment, will provide the mechanistic basis for precision cancer-associated fibroblast-targeted (CAF-targeted) strategies. Such strategies may enhance therapeutic responses and extend survival in advanced and castration-resistant PCa.

Diagnostic and therapeutic strategies targeting CAFs are evolving toward precise subtype stratification, rational combination regimens, and simultaneous multi-target, multi-pathway interventions. Accordingly, systematically delineating CAFs origins, heterogeneity-based subtypes, key signaling axes, and translational advances in PCa will provide a framework and target atlas to guide precision combination therapies.

In this context, we systematically synthesize current evidence on CAF origins, heterogeneity-informed subtypes, and CAF-driven mechanisms that contribute to immune suppression, extracellular matrix (ECM) remodeling, angiogenesis, metabolic support, and therapy resistance in PCa, with an emphasis on insights enabled by emerging single-cell and spatial-omics technologies and mechanistic studies. We further summarize recent translational advances and therapeutic opportunities—highlighting precision CAF targeting, reversible functional reprogramming, rational combination regimens, and localized delivery approaches—to provide a subtype-aware framework and practical target atlas for improving outcomes in high-risk and treatment-refractory PCa.

## 2. Materials and Methods

This review was developed based on a structured literature search followed by focused manual screening. Electronic searches were conducted in PubMed, ScienceDirect, and publisher platforms including Nature and Springer (SpringerLink), from database inception to 15 February 2025 (last search date). Search terms were combined using Boolean operators and included synonyms and related concepts for prostate cancer and cancer-associated fibroblasts, such as “prostate cancer” OR “PCa”, “castration-resistant” OR “CRPC”, “androgen deprivation therapy” OR “ADT”, “cancer-associated fibroblast” OR “CAF”, “tumor microenvironment”, “extracellular matrix”, “immune suppression”, “single-cell”, “spatial”, “heterogeneity”, “plasticity”, and “therapy resistance”. Reference lists of key articles and relevant reviews were also hand-searched to identify additional eligible studies.

References were managed using EndNote 21. Figure 2 was created with BioRender (BioRender.com), and the appropriate license for publication has been secured. This work is a literature-based review and did not generate new datasets or analysis code; therefore, no new code is available for sharing. This review was not preregistered, and no preregistration identifier applies. Generative AI was not used to generate scientific content, extract or analyze data, interpret results, or create figures. ChatGPT-5.2 was used only for superficial language polishing. All scientific content and final wording were verified and approved by the authors.

## 3. Overview of the Prostate Cancer Microenvironment and CAFs

**Figure 1:**
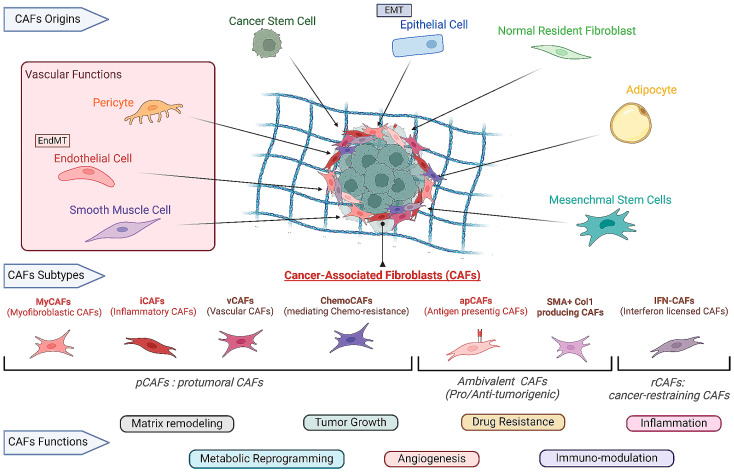
The Origin, Subtypes, and Functions of CAFs. Cancer-associated fibroblasts (CAFs) are highly heterogeneous stromal cells within the TME, originating from various precursors including resident fibroblasts, mesenchymal stem cells, epithelial cells, adipocytes, cancer stem cells, and endothelial cells, as well as through epithelial-to-mesenchymal transition (EMT) and endothelial-to-mesenchymal transition (EndMT). CAFs can be classified into distinct subtypes based on their function and molecular markers, such as MyCAFs, iCAFs, vascular CAFs (vCAF), chemo-resistant CAFs (ChemoCAFs), apCAFs, SMA+ Col1-producing CAFs, and interferon-licensed CAFs (IFN-CAFs). These subtypes exhibit specialized roles, including promoting matrix remodeling, tumor growth, angiogenesis, metabolic reprogramming, and inflammation. Furthermore, CAFs influence drug resistance, immune modulation, and may contribute to both pro-tumorigenic (pCAFs) and anti-tumorigenic (rCAFs) effects depending on their dynamic interactions within the TME. The functional plasticity and origin-specific roles of CAFs emphasize their complexity and their potential as therapeutic targets in cancer treatment. Adapted from Coursier, Diane, and Fernando Calvo. “CAFs vs. TECs: when blood feuds fuel cancer progression, dissemination and therapeutic resistance.” Cellular oncology (Dordrecht, Netherlands). Copyright © 2024 by the authors [19]. This article is an open access article distributed under the terms and conditions of the Creative Commons Attribution (CC BY).

The TME is critical for sustaining tumor growth, invasion, and metastasis. CAFs are the predominant stromal cell type within the TME [9]. CAFs may arise from multiple cellular origins, primarily mesenchymal stem cells; additional sources include cartilage-derived stem cells, mesothelial cells, adipose tissue, endothelial cells, and senescent fibroblasts [11]. During tumor initiation and progression, CAFs expand abnormally relative to normal fibroblasts (NFs) and engage in extensive crosstalk with immune, stromal, and tumor cells [12,13] (Figure 1). They also differ markedly from non-CAFs in gene expression and function [14]. In PCa, pro-tumorigenic factors such as CXCL14 and IL-6 are significantly upregulated in CAFs, whereas stromal caveolin-1 (CAV1) expression is reduced [15,16,17]. Pathway analyses indicate that prostate cancer–associated fibroblasts (PCa-CAFs) are more active in pro-inflammatory and tumor-related signaling (e.g., TGF-β, NF-κB, TNF), suggesting enhanced tumor-promoting capacity. Therefore, PCa-CAFs play pivotal roles in tumor initiation and progression and may influence therapeutic strategies [18]. These CAFs subtypes perform distinct functions in cancer, summarized below:

### 3.1. Immunomodulatory Functions of CAFs

CAFs promote cancer progression and therapy resistance by secreting diverse cytokines and chemokines that modulate immune cell infiltration and activity [20]. CAFs secrete immunosuppressive factors—including checkpoint proteins such as PD-L2 and FASL, as well as vascular endothelial growth factor (VEGF), TGF-β, IL-6, IL-11, and LIF—that dampen antitumor immunity and promote tumor progression [21,22]. Immunosuppressive mediators released by CAFs recruit suppressive myeloid cells and regulatory T cells (Tregs), thereby inhibiting cytotoxic T cell activity and preventing immune recognition and clearance of tumor cells [23]. Regarding T cells, cancer-associated fibroblast–derived (CAF-derived) fibronectin 1 (FN1) binds the CD44 receptor on T cell surfaces. As a key ECM component, FN1 promotes tumor-cell proliferation, invasion, and matrix remodeling, and its expression correlates with poor prognosis and immune cell infiltration across multiple cancers. CD44, in turn, marks cancer stem cells and is strongly associated with unfavorable prognosis, drug resistance, and immune evasion. Together, FN1 and CD44 cooperatively regulate tumor progression and the immune microenvironment [24]. CAF-secreted TGF-β reprograms multiple tumor-associated immune populations by skewing tumor-associated neutrophils (TANs) toward the protumorigenic, immunosuppressive N2 phenotype; polarizing tumor-associated macrophages (TAMs) toward an M2 state that supports tumor growth, metastasis, and tissue remodeling; and suppressing effector T cell function while expanding Tregs, thereby fostering an immunosuppressive milieu that enables immune escape [25]. CAFs recruit monocytes to tumor sites by releasing SDF-1 (via the SDF-1/CXCR4 axis) and CCL2 (monocyte chemoattractant protein-1, MCP-1), inducing their differentiation into M2 macrophages that further promote tumor invasion and metastasis. In turn, factors secreted by M2 macrophages (e.g., IL-10 and TGF-β) activate normal human prostatic fibroblasts (HPFs) into CAFs, forming a pro-tumorigenic positive-feedback loop between CAFs and M2 macrophages [26,27]. In PCa, loss of the HIC1 gene increases tumor-cell secretion of TGF-β, promotes monocyte polarization toward M2 macrophages, and converts NFs into CAFs, establishing a cyclical interaction among tumor cells, CAFs, and M2 macrophages. These findings suggest that HIC1 is a key regulator of the tumor immune microenvironment [28]. CAFs not only deliver inhibitory signals to diverse immune cells but also receive regulatory feedback from other cells. Immune cells within the TME—such as myeloid cells and T and B cells—also signal to CAFs. Myeloid cells upregulate VCAM1, TGF-β, secreted phosphoprotein 1 (SPP1), and the PDGFB ligand–receptor axis that interacts with CAFs, creating a mutually reinforcing positive-feedback loop [24].

### 3.2. The Role of CAFs in ECM Remodeling

ECM remodeling during tumor initiation and progression recapitulates exaggerated wound-healing responses and also plays key roles in organogenesis and tissue repair. Activated CAFs are the principal source of reactive stroma in PCa and, as a key stromal component of the TME, are critical drivers of tumor progression and immune evasion [24,29]. Hallmarks of cancer-associated fibroblast–driven (CAF-driven) ECM remodeling include (i) massive collagen deposition; (ii) reduced α-smooth muscle actin (α-SMA), indicating a shift from myofibroblast toward fibroblast phenotypes; (iii) high vimentin and Tenascin-C expression, marking an actively remodeling ECM; and (iv) markedly increased stromal cell proliferation, evidenced by higher Ki-67 positivity. In the Pro-Cat×JOCK1 and Ubi-Cat×JOCK1 models, WNT signaling robustly activates fibroblasts and generates a dense, collagen-rich ECM. These architectural changes not only provide physical support for tumor growth but also establish a microenvironment with coarse adhesions and pro-proliferative, pro-inflammatory, and pro-metastatic cues, thereby promoting epithelial transformation and adenocarcinoma progression [29,30]. CAF-driven ECM remodeling confers heightened resistance to anticancer agents, with tumor cells exhibiting markedly blunted responses compared with pure tumor organoids. These observations indicate that CAFs remodel the ECM to erect a physical barrier that impedes drug penetration and attenuates therapy-induced cytotoxicity in solid tumors, thereby fostering treatment resistance. In addition, CAFs secrete factors such as WNT16B that mediate resistance to chemotherapy and targeted therapy [31,32,33]. Recent studies show that CAFs express an X-chromosome–encoded androgen receptor (AR) variant that lacks transcriptional activity yet still regulates cellular behavior. Whereas AR primarily promotes proliferation in cancer cells, in CAFs it is closely linked to enhanced motility and invasiveness. By binding the cytoskeletal protein filamin A and activating protease cascades via β1-integrin-dependent signaling, this AR variant triggers ECM remodeling and creates permissive tracks for tumor-cell dissemination [34].

### 3.3. The Role of CAFs in Promoting Angiogenesis

Under physiologic and pathologic conditions, neovessels form to maintain perfusion, remove metabolic waste, and deliver nutrients. Tumor growth is often accompanied by extensive neovascularization. The TME contributes via multiple mechanisms, and CAFs play a pivotal role in peritumoral angiogenesis [23,35,36]. CAFs release proangiogenic cytokines—such as VEGF, stromal cell-derived factor 1 (SDF-1), and fibroblast growth factor (FGF)—that directly stimulate endothelial cells (ECs) and indirectly augment angiogenesis [35]. Elevated CAFs expression of VEGF-A and VEGFR-2 independently predicts biochemical recurrence and risk of progression, and VEGF-A blockade markedly suppresses angiogenesis and tumor growth [37]. In addition, CAFs induce tumor cells to adopt an endothelial-like phenotype and to participate in vessel-wall formation [35]. Beyond direct secretion of proangiogenic factors, CAFs promote angiogenesis via metabolic reprogramming. Mechanistically, secretion of lactate and pyruvate elevates local concentrations, reshapes the metabolic milieu, and drives EC proliferation and angiogenesis [36]. Many CAFs secrete high levels of the chemokine CCL2, which promotes angiogenesis across multiple cancers (e.g., gastric and colorectal) by modulating EC function. In gastric cancer, CCL2 engages ACKR1 on ECs, activates the PI3K–AKT pathway, and enhances EC migration, proliferation, and tube formation, thereby promoting angiogenesis. Similarly, in colorectal cancer, PDPN^+^ CAFs secrete CCL2 to establish a PDPN/CCL2/STAT3 autocrine feedback loop that maintains CAFs heterogeneity and, via paracrine signaling, activates STAT3 in ECs to drive angiogenesis. The STAT3 inhibitor WP1066 disrupts this loop and STAT3 signaling in ECs, thereby suppressing tumor angiogenesis [38,39]. Exposure to chemotherapeutic agents can also switch CAFs to a proangiogenic phenotype. After exposure to cisplatin or 5-fluorouracil (5-FU), CEBPD is markedly upregulated in fibroblasts. CEBPD-high fibroblasts induce SDF4 (Cab45), a CEBPD-regulated secreted calcium-binding protein that acts on human umbilical vein endothelial cells (HUVECs) by engaging CXCR4 to activate ERK1/2 and p38 pathways, upregulate VEGF-D, and promote angiogenesis [40].

## 4. Heterogeneity of CAFs

Single-cell and spatial omics studies increasingly show that CAF subtypes occupy distinct spatial niches [41]. MyCAFs typically localize to peritumoral regions and the invasive front. In contrast, iCAF populations often reside in more distal, immune cell-rich stroma. Vascular CAFs cluster around blood vessels. Antigen-presenting CAFs segregate into tumor-proximal and lymphoid-associated niches [42,43]. However, extensive overlap and dynamic interconversion among these states occur. This suggests that transcriptomic subtypes do not correspond one-to-one with fixed anatomical locations [41]. Tumors at different stages also exhibit heterogeneity in function and phenotype [44].

### 4.1. Spatial Heterogeneity of CAFs Within Tumors

Technologies such as single-cell RNA sequencing and imaging mass cytometry have defined multiple molecular or transcriptional CAF subtypes. These include myCAFs, iCAFs, and apCAFs [43]. However, most of these studies lack spatial positional information. This deficiency hinders understanding of CAF organization within tissues. It also limits insights into how CAFs form local “ecosystems” with tumor and immune cells. Furthermore, it obstructs knowledge on how spatial organizational patterns influence CAF transcriptional states and functions [45].

In 2017, Daniel Öhlund and colleagues identified a spatial-functional framework in a pancreatic ductal adenocarcinoma model. In this framework, myCAF cells are closely adjacent to tumor nests, while iCAF cells locate in the distal stroma. This pattern also appears in skin cancer. There, matrix cancer-associated fibroblasts surround tumor nests. In contrast, immunomodulatory cancer-associated fibroblasts extend into invasive, immune-rich regions. This finding demonstrates that cancer-associated fibroblasts (CAFs) exhibit distinct spatial positional dependence and state plasticity [46]. From a pan-cancer perspective integrated with spatially resolved single-cell analysis, Chenxi Ma and colleagues conducted refined molecular and functional subtyping of CAFs. They identified four stable functional subtypes: inflammatory/immunomodulatory iCAF, ECM/angiogenic mCAF, metabolic meCAF, and proliferative/IFN-I pCAF. They also elucidated subtype-specific transcriptional regulatory networks and metabolic reprogramming features. This work emphasized the common presence of iCAF and mCAF across multiple cancer types. It revealed the division of labor among CAF subtypes in regulating inflammation, remodeling ECM, promoting angiogenesis, and providing metabolic support. Additionally, it highlighted the critical role of iCAF in remodeling myeloid cells and CD8^+^ T cells, as well as promoting T cell exhaustion through signaling axes such as TGFB/CSF1/IL10 and LGALS1–PTPRC–NFATC2. Furthermore, in the context of immunotherapy, they developed an iCAF score. This score serves as a potential biomarker for predicting immune checkpoint blockade (ICB) efficacy. Thus, it directly links CAF molecular phenotypes to immunotherapy response. At the spatial level, CAFs primarily accumulate in the tumor periphery/stromal zone rather than within epithelial compartments. Different molecular subtypes of CAFs, particularly iCAF and mCAF, occupy mutually exclusive “functional zones” within the stroma. They form stable spatial co-localization units with pericytes and endothelial cells. Simultaneously, by altering the spatial distribution of surrounding immune cells (such as neutrophils, Treg cells, and B cells), they collectively shape subtype-specific stromal microenvironments. This suggests a potential lineage evolution pathway from pericytes to iCAF to mCAF [42,47]. Building on this, Liu, Yunhe, and colleagues proposed an analytical framework for “classifying CAFs by spatial neighborhoods.” They utilized various high-throughput spatial omics and multiplex imaging technologies, including CosMx, MERSCOPE, Xenium, Visium, COMET, CODEX, and IMC. This framework adopts the perspective of “tissue structure and spatial neighborhoods.”

Based on their typical histological positions and neighborhood compositions, CAFs can be summarized as follows: tumor-adjacent s1-CAFs, interstitial niche s2-CAFs, myeloid niche s3-CAFs, and lymphoid structure/TLS-associated s4-CAFs. This study employs systematic spatial neighborhood analysis. It demonstrates that the spatial heterogeneity of CAFs manifests not only in differences in histological positions and molecular features but also in profound implications for immune infiltration patterns in the tumor microenvironment, tumor regional partitioning, and clinical prognosis. Specifically, “tumor-adjacent myCAFs” represented by s1-CAFs distribute in close proximity to the tumor bed. They enrich in ECM and THBS1-CD47/TGFβ signaling. These CAFs associate closely with T cell depletion in the tumor bed/edge, immune exclusion structures, and poor prognosis. In contrast, “immune structural organizing CAFs” represented by s4-CAFs associate with TLS. They exhibit high STAT3/HLA-DR expression and drive the aggregation of CCR7^+^ T/B cells. These CAFs correlate significantly with immune activation at the tumor edge and interior, TLS architecture, and better survival. Additionally, s2/s3-CAFs locate in interstitial niches and myeloid-enriched regions. They play important roles in ECM remodeling, inflammatory signaling, and myeloid immune regulation. These CAFs show intermediate or context-dependent associations with T cell states and prognosis across different tumor types [41]. We propose a two-dimensional framework to classify CAFs by spatial position (s1–s4) and functional state (iCAF/mCAF/meCAF/pCAF). s1-like CAFs, which localize to the tumor margin, are ECM-rich and often display mCAF-like immunosuppressive features; these cells may promote immune exclusion and immune evasion. In contrast, TLS-associated CAF subsets resembling s4/apCAF/infCAF, as well as specific immune-supportive iCAF populations, should be identified and preserved to avoid eliminating potentially protective components through nonselective “de-CAF” approaches. In the setting of immunotherapy, rational combination strategies that target iCAFs and key downstream axes (e.g., LGALS1–PTPRC and CXCL12–CXCR4) may enable more precise patient stratification and CAF-informed combination treatment. Collectively, these findings highlight the importance of spatially resolved analyses of CAF heterogeneity for delineating tumor immune architecture and explaining prognostic variation. They also point to opportunities for precision interventions that target defined CAF subsets in specific spatial niches.

### 4.2. CAFs Remodeled into Pro-Drug-Resistant Phenotypes Under Therapeutic Pressure

PCa progression is commonly accompanied by acquired resistance to androgen receptor (AR) pathway inhibitors. Under sustained therapeutic pressure, residual tumor cells can persist by exploiting alternative survival and adaptive cues from the tumor microenvironment (TME), ultimately leading to castration-resistant prostate cancer (CRPC). AR is a central driver of PCa initiation and progression and remains a critical pathway sustaining tumor growth in CRPC. Notably, AR signaling often persists in CRPC and can activate multiple resistance programs, contributing to treatment failure [48,49]. Emerging evidence suggests that AR inhibition can increase ecosystem-level plasticity and heterogeneity, facilitating a gradual shift away from androgen dependence and the emergence of more complex drug-resistant phenotypic lineages under selective pressure [50]. Androgen deprivation therapy (ADT) remains the standard of care for advanced and metastatic PCa. However, most patients eventually develop CRPC [50]. In this setting, cancer-associated fibroblasts (CAFs) are a key cellular population that links therapeutic pressure, TME remodeling, and the consolidation of drug resistance. Their functional heterogeneity and phenotypic interconversion can provide crucial support for the development of CRPC.

Single-cell omics and mechanistic studies indicate that CAF lineages in the CRPC microenvironment differ markedly in composition and functional state from those in hormone-sensitive disease. In CRPC, inflammatory CAFs (iCAFs) are relatively enriched and show elevated expression of PDGFRA, CXCL12, and related markers, together with inflammation-associated transcriptional programs. These iCAFs also occupy central positions in inferred intercellular communication networks [51]. Notably, CRPC-associated iCAFs may reshape the local AR signaling milieu via steroid metabolism–related enzymes (e.g., HSD17B2) while secreting pro-migratory and pro-invasive factors (e.g., ITGBL1), thereby promoting tumor cell invasiveness and a castration-resistant phenotype. In addition, the antiandrogen enzalutamide (ENZ) can further upregulate this axis, suggesting that iCAFs may function as therapy-induced, drug-resistance–supporting nodes within the microenvironment [51]. In addition to the emergence of a pro–drug-resistant iCAF state, therapeutic pressure can drive phenotypic transitions in CAFs, generating myofibroblastic CAF (myCAF) subpopulations with stronger resistance-promoting activity. Under basal conditions, AR activity in CAFs can attenuate responsiveness to TGF-β signaling by suppressing TGF-β receptor expression. Following androgen deprivation therapy (ADT), inhibition of CAF AR increases TGFBR1 expression, heightening TGF-β sensitivity. This response induces SOX4 transcription via SMAD signaling and cooperates with the SWI/SNF chromatin-remodeling complex to promote the transition of iCAFs into SPP1^+^ myCAFs [8]. The resulting SPP1^+^ myCAFs express and secrete high levels of SPP1, which engages integrin receptors on tumor cells and activates the ERK/MAPK pathway, thereby increasing resistance to antiandrogen therapies (e.g., enzalutamide, ENZ). Accordingly, blocking TGF-β signaling to prevent myCAF differentiation, targeting SPP1 to disrupt CAF–tumor cell crosstalk, or inhibiting downstream ERK/MAPK signaling can restore sensitivity to ADT. These observations support the TGF-β–SPP1–ERK axis as a rational target for combination interventions [8].

Progression to castration-resistant prostate cancer (CRPC) generally reflects a more aggressive disease state. This is particularly evident in metastatic CRPC (mCRPC), which is associated with shorter overall survival and is rarely curable with currently available therapies [52]. Recent studies have further developed the resistance-promoting myCAF model by addressing both induction and maintenance. TGF-β1 can drive iCAFs and intermediate CAF states toward a myCAF phenotype. In turn, myCAFs typically exhibit low AR expression, increased motility, and enhanced matrix contractility, and can promote tumor proliferation via secretion of pro-inflammatory mediators. Mechanistically, the NF-κB–TGF-β1–YAP1 axis has been implicated in both the establishment and homeostatic maintenance of the myCAF state; co-inhibition of TGF-β1 and YAP1 partially reverses myCAF activation and increases tumor-cell sensitivity to enzalutamide (ENZ) [53]. In addition, molecular markers such as CTHRC1 help define the functional features of resistance-promoting myCAFs. CTHRC1 is predominantly expressed in myCAFs and is associated with TGF-β pathway activation, which may promote polarization of tumor-associated macrophages (TAMs). CTHRC1 may also modulate AR signaling via CCN2/CAV1/AR-related pathways. Clinically, tumors with high CTHRC1 expression show reduced responses to androgen deprivation therapy (ADT) and are more likely to progress to an aggressive castration-resistant phenotype [54]. Collectively, these data suggest that myCAF functional subtypes characterized by SPP1 and CTHRC1 contribute to drug resistance and malignant progression during CRPC evolution. However, their clinical translation—and the robustness of associated stratification biomarkers—requires further validation [53,54].

Earlier animal studies suggest that ADT/castration-induced microenvironmental stress can establish a positive feedback loop involving hypoxia, TGF-β signaling, and myCAFs, coupled to immune reprogramming. Castration promotes myCAF accumulation in residual tumor regions and increases CXCL13 secretion, which drives B-cell infiltration and a protumor immune milieu. Conversely, inhibition of TGF-β signaling suppresses myCAF activation, reduces CXCL13 expression and B-cell infiltration, and delays CRPC recurrence. Because fibroblasts are a major source of TGF-β, this circuit may be self-reinforcing [55]. Clinically, tumors after ADT may progress through regression, selection of drug-resistant clones, and subsequent CRPC regrowth [10]. During this process, drug-resistant stem/progenitor-like epithelial populations can stimulate myCAFs to produce CCL2 via soluble mediators (e.g., TNF), thereby recruiting CCR2^+^ tumor-associated macrophages (TAMs). The resulting myeloid infiltration can promote immunosuppression and immune evasion, facilitating CRPC regrowth and disease progression [10]. In summary, therapeutic pressure acts not only on tumor cells but also on the TME by remodeling CAF states (e.g., iCAF activation and iCAF-to-myCAF transition) and rewiring their interactions with immune networks. This remodeling can generate a microenvironmental ecosystem that promotes antiandrogen resistance and CRPC progression. Accordingly, combination strategies that target key nodes—such as TGF-β, SPP1/CTHRC1, ERK/MAPK, and YAP1—may offer mechanistically grounded approaches to constrain the evolution of drug resistance [8,53,54,55].

Across multiple cancer types, CAF subsets with antigen-presenting capacity have been shown to activate CD4^+^ T cells and modulate tumor immunity [56,57]. In 2024, Wang and colleagues isolated primary CAFs from resected prostate cancer specimens from patients with or without prior androgen deprivation therapy (ADT) and performed SMART-based transcriptome sequencing. They identified 281 differentially expressed genes; 147 were downregulated in post-ADT CAFs and were enriched for antigen processing and presentation, the MHC class II protein complex, and related GO/KEGG pathways. These results are consistent with an overall attenuation of antigen presentation–associated programs in CAFs after castration therapy. Using prostate cancer single-cell sequencing datasets, the authors curated 431 antigen processing/presentation–related genes and applied NMF clustering and pseudotime analysis to CAFs. This analysis identified an antigen processing and presentation–related CAF subtype (APPCAF), represented by the CTSK^+^MRC2^+^ CAF-C1 cluster. APPCAFs showed extensive ligand–receptor interactions with epithelial cells and T cells, were closely associated with inflammatory CAF features, and resembled apCAFs reported in other tumors (e.g., pancreatic cancer). Under in vitro ADT-mimicking conditions, APPCAF signature genes (THBS2, COL5A1, and MARCKS) were further upregulated in CAFs, whereas DPT was modestly downregulated. In early CD4^+^ T-cell activation assays, pre-castration CAFs induced upregulation of CD25 and CD69 in OVA-specific CD4^+^ T cells, whereas post-castration CAFs largely lost this capacity. Functionally, these results support that ADT weakens CAF-mediated antigen presentation and immune priming. Taken together, the authors propose that androgen withdrawal reprograms not only tumor cells but also the stroma. By downregulating MHC class II and antigen-processing/presentation pathways, it may shift APPCAF/apCAF-like CAFs toward a state less capable of activating CD4^+^ T cells and more permissive to immune escape, representing a form of therapy-induced stromal reprogramming during the evolution of castration resistance. Accordingly, targeted modulation of APPCAF/apCAF-like CAFs may offer an entry point to optimize ADT–immunotherapy combination strategies [58].

## 5. CAFs Function Subclusters in PCa

CAFs are heterogeneous and comprise multiple subtypes with distinct origins and functions. These subtypes perform distinct roles in PCa [35]. However, CAFs constitute a highly heterogeneous, functionally plastic population that is dynamically regulated by the microenvironment. Depending on context, they can promote or suppress tumor growth. These context-dependent roles indicate functional specificity among CAFs subtypes. Therefore, selective identification and targeting of defined subtypes are crucial for antitumor efficacy [59,60]. Pan-cancer single-cell and spatial omics studies consistently support classifying CAFs into four major functional modules—myCAF, iCAF, apCAF, and vCAF—alongside a limited number of cancer-specific subtypes (e.g., prostate cancer–specific scRNA-seq–defined clusters). Together, these categories capture the defining features of function-dominant CAF states [42,43,61,62] (Table 1). Elucidating the protumor roles of CAFs subtypes in PCa is essential for developing precision CAF-targeted or differentiation-inducing strategies [63].

### 5.1. Myofibroblastic CAFs (myCAFs)

In PCa, myCAFs constitute a principal CAFs subtype. Cancerous stroma is predominantly myCAF-rich, whereas normal prostatic stroma is largely smooth muscle [85]. Evidence indicates multiple origins for myCAFs. Paracrine factors from tumor epithelial cells (e.g., TGF-β, FGF) drive adjacent fibroblasts or mesenchymal stem cells to differentiate into myofibroblasts. myCAFs may also arise through EMT and endothelial-to-mesenchymal transition (EndMT), with TGF-β as a key regulator [64,65,66]. myCAFs promote tumor cell proliferation and migration through ECM remodeling and signaling. The PCa microenvironment undergoes continual remodeling that sustains glandular and tumor architecture while driving excessive ECM accumulation, tissue disorganization, and functional decline—hallmarks of a dysregulated wound-healing response [65,66,85]. myCAFs exhibit canonical myofibroblast features. These include expression of fibroblast-associated genes ACTA2 (encoding α-smooth muscle actin; α-SMA) and TAGLN (transgelin); ECM-related genes MYL9 and TPM1 (isoform specificity to be determined); and high levels of collagen genes such as COL1A1 and COL1A2. These features indicate that myCAFs contribute to ECM remodeling, collagen metabolism, smooth-muscle contraction, and cell adhesion—processes central to tumor progression and metastasis [67]. myCAFs coexpress the matrix metalloproteinases MMP2 and MMP14 and secrete proangiogenic growth factors, creating an active stromal milieu that remodels pathologic tissue, stimulates tumor growth and dissemination, and facilitates tumor cell migration across ECM components such as collagen, fibronectin, and hyaluronan [67,86,87,88].

Crosstalk between stromal cells and the tumor immune microenvironment (TIME) is widely recognized as a key driver of PCa progression [89]. Monoamine oxidase A (MAOA) is a mitochondrial enzyme; its upregulation in stromal compartments has been linked to prostate tumorigenesis. In 2025, Zhao and colleagues used single-cell transcriptomic analysis to show that MAOA expression is markedly higher in myCAFs than in other CAFs subtypes. myCAFs also express high levels of signature genes—including ACTA2, RGS5, and MYH11—that strongly correlate with MAOA, suggesting that MAOA supports the myCAF phenotype and preserves canonical features. In addition, MAOA promotes the generation of reactive oxygen species (ROS). Elevated ROS suppresses WNT5A in CAFs; WNT5A loss further diminishes the ability of CAFs to activate CD8^+^ T cells. Consequently, sustained ROS driven by MAOA pushes myCAFs toward an immunosuppressive state, dampening T cell activity and facilitating tumor immune evasion. MAOA inhibition markedly reduces α-SMA, MYH11, and other myCAF markers in CAFs. Concurrently, CAFs secrete more WNT5A, which activates Ca^2+^–NFATC1 signaling in CD8^+^ T cells, increases expression of cytolytic factors (GZMB, IFN-γ), augments tumor cell killing, and strengthens antitumor immunity [68]. Taken together, these findings indicate that MAOA expressed by myCAFs is crucial for maintaining the myCAF phenotype and regulating their immunosuppressive functions, thereby shaping crosstalk with immune cells.

### 5.2. Inflammatory CAFs (iCAFs)

iCAFs can arise from multiple precursors, including resident fibroblasts, mesenchymal stem cells (MSCs), endothelial cells via EndMT, adipocytes, and bone marrow–derived progenitors. Cytokines derived from PCa epithelial cells are key drivers of the iCAF state. Common inducers include IL-1, TGF-β1, and CXCL16, as well as lactate produced by tumor metabolism and oxidative stress; together these stimuli elevate reactive ROS and drive CAFs activation toward an iCAF state. Hallmarks of iCAFs include high expression of inflammatory mediators (e.g., IL-6, IL-8, IL-1β, CSF2, CSF3) and chemotactic factors (PDGFRA, LIF, CXCL1, CXCL12, CXCL14, CCL2), together with increased components of the complement cascade (e.g., C3, C7, CFD). These features promote macrophage recruitment, angiogenesis, and immune-cell migration, sustain an inflammatory microenvironment, and correlate with tumor progression [13,51,69].

CXCL14 produced by iCAFs promotes protumor processes, including monocyte recruitment, M2 macrophage polarization, and angiogenesis. Augsten and colleagues found that nitric oxide synthase 1 (NOS1) is upregulated in CXCL14^+^ CAFs and is associated with oxidative stress, implicating NOS1 in the regulation of cell fate and metabolism. In CXCL14-induced CAFs, the NRF2 and HIF-1α signaling pathways are activated. NRF2 is the master regulator of the oxidative-stress response, enhancing antioxidant defenses and promoting CAFs survival and function [90,91]. HIF-1α regulates metabolism and induces programs that enable adaptation to hypoxia in the TME [92,93]. Accordingly, CXCL14-induced iCAFs sustain protumor activity by enhancing their growth and metabolic adaptability and by remodeling the TME—through immunosuppression and promotion of metastasis.

Chronic inflammation drives PCa initiation and progression, in part by establishing a protumorigenic milieu within the TME. Inflammation-related pathways (e.g., NF-κB, STAT3, PI3K–AKT) regulate cell survival, apoptosis resistance, EMT, and angiogenesis. Multiple therapies (ADT, chemotherapy, radiotherapy) can induce inflammatory signaling, which enhances tumor-cell drug resistance; thus, inflammatory signaling is a critical target in preventing PCa progression and treatment failure [71]. In 2021, using coculture experiments, Linda L. Tran and colleagues showed that human MSCs stimulated with IL-1α differentiate into iCAFs that robustly secrete IL-6, CXCL1, LIF, and PTGS2 (COX-2). In PCa, these factors activate STAT3, NF-κB, and androgen receptor splice variant 7 (AR-V7) signaling, thereby promoting tumor-cell survival, immune evasion, and androgen desensitization, and markedly increasing proliferation. RNA-seq and quantitative PCR (qPCR) likewise showed a pronounced increase in the transcription factor ELF3. Knockdown of ELF3 markedly attenuated the pro-proliferative effect, indicating that ELF3 is a key driver of iCAF differentiation and of an ELF3-regulated inflammatory paracrine program (IL-6, CXCL1, LIF) that contributes to pro-PCa activity. These data identify ELF3 as a molecular hub of iCAF formation and a potential target for TME-directed intervention [69]. In 2024, Qijun Wo and colleagues integrated single-cell analyses, bioinformatic screening, and animal experiments and found that the key gene HHIP is downregulated in PCa tissues and in CAFs. HHIP upregulation suppressed CAFs secretion of inflammatory factors (e.g., IL-6, TGF-β, IL-1β), whereas HHIP downregulation enhanced inflammatory-factor secretion and stabilized the CAFs phenotype, with increased α-SMA expression. Moreover, HHIP upregulation reduced α-SMA in CAFs, suggesting partial reversion toward a normal fibroblast phenotype. HHIP also inhibited JAK1/STAT3 phosphorylation, lowering p-JAK1 and p-STAT3, consistent with suppression of downstream proinflammatory mediators via reduced JAK1/STAT3 activation [70]. Collectively, these findings indicate that HHIP in CAFs negatively regulates inflammatory cytokine secretion and may help maintain a noninflammatory CAFs state, offering a potential target to modulate TME inflammation and restrain tumor progression.

### 5.3. Antigen-Presenting CAFs (apCAFs)

Research on myCAFs and iCAFs is relatively advanced, and the role of CAFs in the tumor immune microenvironment has garnered extensive attention. Consequently, apCAFs are emerging as a research focus. Emerging evidence suggests that apCAFs arise mainly from mesothelial cells via MMT driven by IL-1–NF-κB and TGF-β–SMAD signaling [72]. apCAFs constitute a functionally specialized CAFs subpopulation defined by antigen-presentation molecules such as MHC-II and CD74; they often coexpress mesothelial and CAFs structural markers [73]. In PCa, apCAFs—fibroblast subsets expressing MHC-II—primarily promote immune evasion by altering immune-cell function, suppressing antigen presentation, and enhancing tumor immune tolerance. These mechanisms enable PCa to evade host immune surveillance, facilitating continued growth and metastasis [58].

Recent prostate cancer single-cell and spatial transcriptomic analyses (e.g., by Han Lin and colleagues) classified CAFs into iCAF, myCAF, and apCAF states, and identified C11_HLA-DRA as a representative apCAF cluster. This cluster was characterized by an antigen-presentation signature (e.g., HLA-DRA, CD74, and MHC class II genes) and was associated with PSCA-linked spatial patterns, thereby positioning apCAFs as a functionally defined CAF state within prostate tumor microenvironment heterogeneity frameworks. Using spatial mapping and cell–cell communication inference, the authors argued that apCAFs are not only transcriptionally defined but may also occupy discrete niches in proximity to PSCA^+^ tumor cells. Spatial transcriptomics indicated that regions enriched for PSCA^+^ tumor cells also showed enrichment of the C11_HLA-DRA cluster, consistent with a potential local communication hub. In CellChat-based analyses, predicted interactions between PSCA^+^ cells and C11_HLA-DRA were among the strongest, with PTN–NCL highlighted as a representative axis (alongside MDK–LRP1 and IFNG–IFNGR1/2). These predicted signals were interpreted in the context of angiogenesis (e.g., PTN–NCL), immunoregulatory programs (e.g., HLA-DRA–associated antigen-presentation machinery), and tumor-progression–related pathways, leading the authors to propose that apCAFs contribute to tumor–stroma crosstalk and may favor immune-excluded (“cold”) microenvironments [94]. Similarly, in pancreatic ductal adenocarcinoma (PDAC), Elyada and colleagues defined apCAFs by high MHC class II and Cd74 expression and strengthened the evidence base through in vivo, histologic, and functional assays. They confirmed the presence of double-positive cells expressing fibroblast markers together with MHC class II/CD74 in tumor tissue. Using the OTII (OVA-specific) CD4^+^ T-cell system, they further showed that sorted MHCII^+^ CAFs could induce early T-cell activation markers under antigen-loading conditions, providing functional support for antigen presentation by CAFs. They also raised immunosuppression-related hypotheses based on the reported lack of co-stimulatory molecules [43]. In prostate cancer, HLA-DRA^+^ apCAFs have been proposed as potential mediators of immune escape. However, the prostate cancer analysis appears to rely largely on reanalysis of public datasets and is constrained by limited experimental validation, sample size, and analytical parameter choices; therefore, these conclusions warrant further confirmation in independent cohorts and mechanistic experiments.

### 5.4. Metabolic CAFs (meCAFs)

Metabolism plays a critical role in the development and progression of PCa. Recent studies indicate that metabolic reprogramming is a core mechanism driving PCa initiation, progression, metastasis, and drug resistance [95]. meCAFs can activate aerobic glycolysis, generating large amounts of high-energy metabolites (e.g., lactate and pyruvate); these products are shuttled back to tumor cells via the monocarboxylate transporter (MCT) “lactate shuttle,” fueling efficient mitochondrial oxidative phosphorylation (OXPHOS)—an important function of meCAFs within tumor tissues [96]. meCAFs arise primarily from local fibroblasts and display distinctive functional origins; influenced by oncogenic signals from cancer cells, epigenetic regulation, and metabolic-enzyme expression, they exhibit metabolic activities and pro-tumor functions that differ from conventional CAFs. Among the drivers, intracellular oncogenic signals (such as RAS, TGF-β, and NF-κB) induce neighboring NFs to convert into meCAFs through oxidative stress, and hypoxia triggers HIF-1α–dependent epigenetic changes in NFs, driving their transition to highly glycolytic meCAFs [74,75,76].

PCa is a malignancy that depends heavily on support from the TME [97], and CAFs, as metabolic symbiotic partners, engage in highly complex bidirectional interactions with tumor cells [98]. A defining feature of meCAFs is high-level lactate secretion. Lactate is not only abundant in the TME but also essential for PCa-cell metabolism, helping to explain the metabolic coupling between PCa cells and CAFs [76]. In 2012, Fiaschi and colleagues delineated the metabolic crosstalk between PCa cells and CAFs. Upon tumor-derived induction, CAFs adopt a Warburg-like phenotype—maintaining aerobic glycolysis, producing large amounts of lactate, and exporting it via upregulated MCT4. This transition is driven by ROS-dependent stabilization of HIF-1α and is accompanied by downregulation of SIRT3 (an NAD^+^-dependent deacetylase) and increased acetylation of SOD2 (superoxide dismutase). In turn, PCa cells upregulate the lactate importer MCT1, take up CAF-derived lactate to fuel the tricarboxylic acid (TCA) cycle and biosynthesis, and thereby secure energy and anabolic precursors in a low-glucose, high-stress TME, promoting proliferation and progression. This establishes a metabolic symbiosis dependent on a lactate shuttle. This study was the first to clearly reveal a bidirectional metabolic-reprogramming mechanism between CAFs and PCa cells mediated by the lactate shuttle [77]. In 2019, Ippolito and colleagues showed that CAF-derived lactate reprograms PCa cells and drives a glycolysis-to-OXPHOSshift. Lactate is converted to pyruvate by LDHB, altering the NAD^+^/NADH ratio and activating the SIRT1/PGC-1α axis, which in turn induces mitochondrial biogenesis and metabolic upregulation. In addition, PCa cells can directly acquire functional mitochondria from CAFs via structures such as intercellular bridges, further augmenting OXPHOS capacity and the efficiency of lactate utilization. This metabolic reprogramming is accompanied by accumulation of succinate and fumarate in the tricarboxylic acid cycle, which inhibits prolyl hydroxylases (PHDs) and stabilizes HIF-1α, thereby inducing EMT and increasing cellular invasiveness [81]. These findings highlight the central roles of lactate metabolism and mitochondrial dynamics in PCa progression and provide a rationale for therapies targeting tumor–stroma metabolic symbiosis.

Beyond increasing lactate production through reprogramming of their own glucose metabolism—a reverse Warburg effect—CAFs also broadly regulate amino-acid and lipid metabolism, thereby providing metabolic support to tumor cells [80]. In 2022, Ippolito and colleagues showed that CAF-derived lactate is imported by PCa cells via MCT1. After entry into the TCA cycle, lactate carbon is converted to citrate and then to acetyl-CoA by ATP-citrate lyase (ACLY), thereby driving fatty-acid synthesis by enzymes such as acetyl-CoA carboxylase (ACC) and fatty acid synthase (FASN), promoting fatty-acid and cholesterol synthesis and ultimately lipid-droplet accumulation. Analyses of clinical specimens showed that high-Gleason tumors display elevated lactate, lipid metabolites, and MCT1 expression, indicating a close association between lactate metabolism and tumor aggressiveness. Stable-isotope and radiolabeling experiments traced lactate carbon extensively into lipids, and this flux was effectively blocked by MCT1 inhibition [78]. Together, these findings delineate a lactate-fueled metabolic axis (MCT1–ACLY–lipid-droplet formation) through which CAFs bolster tumor-cell lipid synthesis, revealing a key mechanism of metabolic adaptation in PCa.

In recent years, amino acid metabolism has become a major focus of cancer-associated fibroblast-mediated (CAF-mediated) metabolic remodeling in tumors. Through multiple mechanisms, CAFs regulate amino acid synthesis, transport, and reuse, thereby providing essential metabolic support for rapid proliferation, invasive migration, and stress tolerance of tumor cells [98]. In 2018, Mishra and colleagues reported that, within the PCa microenvironment, CAFs epigenetically silence RASAL3, a negative regulator of RAS signaling, thereby activating RAS and initiating macropinocytosis. CAFs then take up large amounts of exogenous albumin, which is degraded in lysosomes to generate glutamine. The newly synthesized glutamine is secreted into the TME to supply metabolic substrates for PCa epithelial cells. Tumor cells upregulate transporters such as SLC1A5 to import glutamine and channel it into energy production (tricarboxylic acid, TCA, cycle), antioxidant defense, and nucleotide biosynthesis. Moreover, glutamine activates the mTOR–FOXM1 pathway to induce neuroendocrine (NE) differentiation, thereby enhancing resistance to ADT [79]. Beyond synthesizing glutamine to promote PCa progression, CAFs also generate other key nitrogen-donating amino acids—such as aspartate and asparagine—for tumor-cell utilization. Subsequent work by Li and colleagues showed that p62-deficient CAFs maintain metabolic adaptability under glutamine-deprived conditions by stabilizing activating transcription factor 4 (ATF4). Loss of p62 blocks p62-mediated, ubiquitin-dependent degradation of ATF4, thereby increasing ATF4 stability and upregulating asparagine synthetase (ASNS) and pyruvate carboxylase (PC). ASNS converts aspartate to asparagine, whereas PC converts pyruvate to oxaloacetate, supplying substrates for the TCA cycle. These metabolic changes enable p62-deficient CAFs to synthesize and secrete aspartate and asparagine, providing essential nitrogen for PCa cells and supporting sustained growth under glutamine-limited conditions [80]. Looking ahead, deeper investigation of additional amino-acid pathways in CAFs suggests that targeting CAFs amino-acid metabolism may offer an effective anticancer strategy.

### 5.5. Other Less Prevalent CAFs Subtypes

Diverse origins and activating cues, microenvironmental differences, and epigenetic plasticity underpin the marked functional heterogeneity of CAFs, which comprise a highly plastic cell population [99]. Single-cell RNA sequencing (scRNA-seq) enables unbiased deconvolution and high-resolution subtyping of CAFs [23]. Beyond the well-studied subtypes, PCa harbors additional emerging CAFs subsets with potential clinical relevance.

PCa exhibits marked heterogeneity across disease stages, particularly in CRPC, where cancer cells evade therapy through multiple mechanisms. Although approved treatments (e.g., ENZ, abiraterone) provide survival benefits, CRPC still gradually develops resistance and lacks a standardized treatment regimen [100,101]. In 2024, Zhao and colleagues integrated scRNA-seq with spatial transcriptomics to track changes in cell states and the microenvironment across disease stages and to map the spatial distribution of PCa cells. The study identified a myofibroblast-like CAFs population (mCAFs) that accumulates progressively during PCa progression and correlates with higher Gleason scores and poorer prognosis. mCAFs display relatively low α-SMA expression yet play pivotal roles in extracellular-matrix remodeling, angiogenesis, skeletal development, and EMT. They influence cancer progression and drug resistance via immunosuppression and NE transdifferentiation, which is tightly linked to enzalutamide resistance. mCAFs also show spatial colocalization with immune cells (e.g., SPP1^+^ macrophages, Treg cells), together forming an immunosuppressive niche that enhances tumor immune evasion. In addition, mCAFs may further exacerbate resistance by promoting NE transdifferentiation [83]. In 2023, Pan and colleagues used scRNA-seq to identify two major CAF subtypes—CAF-C0 (α-SMA^+^/CAV1^+^) and CAF-C1 (FN1^+^/FAP^+^)—with distinct molecular and functional profiles. CAF-C0 predominantly expresses α-SMA and CAV1; genes related to microvascular development, such as MYH1, MCAM, and RGS5, are also highly expressed, indicating a close association with microvasculature formation. By contrast, CAF-C1 shows higher expression of collagen molecules (e.g., COL1A1, COL3A1), FN1, and FAP, functioning mainly in ECM remodeling. Immunofluorescence analysis of PCa specimens showed that CAF-C0 is most abundant in HSPC, whereas CAF-C1 predominates in CRPC. Trajectory analysis indicated that CAF-C0 occupies an early stage of subtype transition, whereas CAF-C1 lies at a late stage, suggesting distinct roles during PCa progression. High FN1 and FAP expression in CAF-C1 marks poor prognosis in PCa and further implicates this subtype in therapeutic resistance. Accordingly, CAF-C1 is considered a key driver of therapeutic resistance and malignant progression in PCa [62]. These discoveries provide new avenues for developing CAF-targeted therapies for PCa—particularly at the CRPC stage—which may help mitigate resistance, control metastasis, improve quality of life, extend survival, and enable personalized treatment.

Single-cell transcriptomic analyses showed that this subset is present in both murine and human PCa and is enriched for gene programs related to mineral absorption, iron metabolism, and ferroptosis. ferroptosis-associated cancer-associated fibroblasts (FerroCAFs) show high expression of the surface marker CD155 (PVR; poliovirus receptor) and of Hmox1, and their intracellular ferrous iron (Fe^2+^) content is markedly higher than in other CAFs subgroups. Mechanistically, Hmox1 catalyzes heme degradation to release Fe^2+^, which activates the iron-dependent demethylase Kdm6b, lowers H3K27me2/3, and upregulates myeloid cell–associated factor (MASP) genes such as CCL2, CSF1, CXCL1, and IL6. Functionally, FerroCAFs recruit and polarize immunosuppressive myeloid cells by secreting MASPs, thereby weakening CD8^+^ T cell cytotoxicity. The FerroCAF fraction increases with higher Gleason grade, remains detectable in metastatic castration-resistant disease, and correlates with an immune-cold milieu and poor prognosis. Inhibition of Hmox1 or Kdm6b reduces iron load and immunosuppressive-factor expression, improves the immune microenvironment, and suppresses tumor growth [82]. Therefore, blocking the immunosuppressive functions of FerroCAFs may enhance the efficacy of immune-checkpoint inhibitors such as anti–PD-1/PD-L1 antibodies.

In 2025, Chen Ding and colleagues used scRNA-seq to profile CAFs heterogeneity and function in PCa. They identified four major CAFs subtypes within the TME: C0 IER2^+^, C1 ABCA8^+^, C2 ABI3BP^+^, and C3 MEOX2^+^. The C1 ABCA8^+^ subtype exhibits increased proliferative activity and is implicated in tumor growth and metastasis. NEFH^+^ PCa cells secrete pleiotrophin (PTN), which activates the nucleolin (NCL) receptor on fibroblasts, thereby promoting their conversion into CAFs. The PTN–NCL interaction regulates intercellular signaling strength, cell migration, and immune-microenvironment modulation. The study proposed and validated a NEFH^+^ tumor cell–PTN–NCL–CAFs conversion axis in PCa, underscoring the central role of the C1 ABCA8^+^ subtype in tumor progression. The study also found distinct transcription-factor programs across CAFs subpopulations; in C1 ABCA8^+^ cells, NFAT5 was markedly upregulated, suggesting a candidate driver of this subtype. To probe the protumor properties of C1 ABCA8^+^ fibroblasts and the role of NFAT5, the authors knocked down NFAT5 in PCa cell lines (LNCaP, 22Rv1). NFAT5 loss reduced cell viability, clonogenic growth, and migratory and invasive capacities. These findings support a model in which the C1 ABCA8^+^ fibroblast program—via NFAT5 and related regulators—promotes PCa-cell proliferation and metastasis [84].

## 6. Pro-Tumor Pathways and Molecular Hubs in CAFs

**Figure 2:**
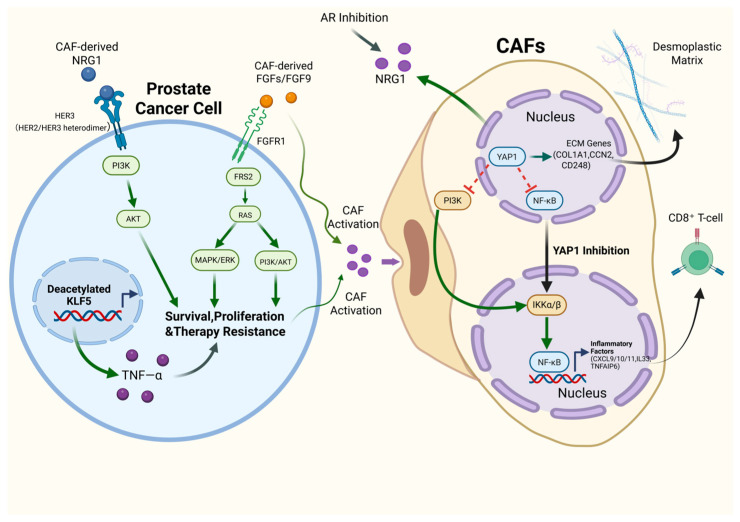
Illustrations of three CAF-centered, actionable hubs that support prostate cancer progression and therapy resistance under AR pathway inhibition: (3) CAF-derived NRG1 activates tumor-cell HER3 (HER2/HER3 heterodimer) to trigger PI3K–AKT, promoting survival and antiandrogen resistance (targetable via anti-NRG1/anti-HER3 or PI3K/AKT blockade); (2) CAF-derived FGFs/FGF9 signal through tumor FGFR1–FRS2, branching to RAS–MAPK/ERK and PI3K–AKT to drive proliferation/survival and resistance (targetable at FGFR or downstream nodes such as FRS2/MAPK/PI3K); and a tumor deacetylated KLF5→TNF-α module reinforces CAF activation. In CAFs, (1) nuclear YAP1 induces ECM genes (e.g., COL1A1, CCN2, CD248) and desmoplastic matrix formation while suppressing IKKα/β–NF-κB; YAP1 inhibition releases NF-κB signaling to upregulate inflammatory/chemotactic factors (CXCL9/10/11, IL33, TNFAIP6), enhancing CD8^+^ T-cell recruitment/activation, supporting YAP1 as a stromal reprogramming target (often in combination with immunotherapy). Figures were created with BioRender.com and publication rights have been obtained.

Clinically, most patients with metastatic prostate cancer eventually progress to castration-resistant prostate cancer (CRPC) under sustained androgen deprivation and next-generation androgen receptor (AR) pathway inhibition, and many subsequently develop secondary resistance to taxane chemotherapy and immunotherapy [50,102,103]. Traditionally, therapeutic resistance has been attributed mainly to tumor-intrinsic mechanisms, including genetic alterations, epigenetic remodeling, and lineage plasticity (e.g., neuroendocrine prostate cancer, NEPC) [50,104]. However, accumulating transcriptomic, single-cell, and spatial omics evidence suggests that cancer-associated fibroblasts (CAFs) are not passive background stroma. Instead, therapeutic pressure can reprogram CAFs into pro-tumor functional modules that promote tumor survival, adaptation, and treatment resistance through soluble signaling, metabolic reprogramming, extracellular matrix (ECM) remodeling, and immune modulation. In prostate cancer, these CAF programs converge on multiple actionable nodes that drive disease progression [8,78,105,106]. Below, we summarize experimental and preclinical evidence supporting these CAF-centered nodes and discuss how they can be leveraged as therapeutic entry points for stromal-targeted combination strategies in prostate cancer (Figure 2).

### 6.1. YAP1

Across solid tumors, the Hippo-pathway effector YAP1 has emerged as a central regulator of CAF activation and functional reprogramming. Tumor-conditioned fibroblasts increase actin stress fibers and extracellular matrix (ECM) production, thereby elevating matrix stiffness and cytoskeletal tension. These mechanical cues can promote YAP1 nuclear translocation via RhoA–FAK–Src signaling, which in turn induces matrix-remodeling and myocontractile programs (e.g., ACTA2/α-SMA, collagens, and fibronectin), enabling CAFs to acquire stable pro-tumor functions such as ECM remodeling, matrix contraction, and metabolic support [107,108]. In parallel, YAP1-dependent programs can enhance CAF proliferation and invasiveness through secretion of cytokines, chemokines, and exosomes, remodeling of collagen networks, and activation of integrin–FAK signaling. YAP1-active CAFs may also foster an immunosuppressive microenvironment that limits CD8^+^ T-cell infiltration and reduces responsiveness to immune checkpoint blockade [109,110,111]. Clinicopathologic studies report elevated nuclear YAP1 in the tumor stroma of more aggressive prostate cancers, including cases with lymph node metastasis or seminal vesicle invasion. These observations suggest that YAP1^+^ CAFs may contribute to disease progression and treatment response, providing a rationale to examine the YAP1–CAF axis in prostate cancer [112,113].

In the prostate cancer microenvironment, multi-level evidence supports a YAP1-centered trajectory of CAF evolution that can be conceptualized in three steps: origin (normal fibroblasts, NFs → CAFs), maturation (early → late myCAFs), and functional reprogramming (ECM-CAFs → Lym-CAFs). At the origin stage, Tianyu Shen and colleagues reported that YAP1 promotes the transition from NFs to CAFs and helps sustain CAF pro-tumor programs. Relative to benign prostatic hyperplasia, stromal YAP1, FAP, and α-SMA levels were higher in prostate cancer, with substantial spatial overlap between YAP1 staining and the CAF marker FAP. Tumors with high stromal YAP1 also showed an increased abundance of α-SMA^+^ CAFs. Stromal nuclear YAP1 positivity was associated with adverse clinicopathologic features, including higher Gleason score, elevated PSA, advanced T stage, and lymph node metastasis or seminal vesicle invasion. In vitro, CAFs expressed higher YAP1, FAP, and α-SMA at both mRNA and protein levels than NFs. YAP1 knockdown in CAFs reduced FAP and α-SMA expression, suppressed proliferation, and partially attenuated CAF-like characteristics. Conversely, YAP1 overexpression in NFs induced FAP and α-SMA expression and promoted a CAF-like phenotype with increased proliferative capacity. Conditioned media from YAP1-high fibroblasts (primary CAFs or YAP1-overexpressing NFs) enhanced proliferation and invasion of multiple prostate cancer cell lines and promoted an EMT-like shift, characterized by reduced E-cadherin and increased N-cadherin and vimentin. In contrast, YAP1 knockdown in CAFs weakened these pro-tumor effects. Mechanistically, YAP1 forms a complex with TEAD1 and can activate SRC transcription, increasing SRC expression and cytoskeleton-associated programs (e.g., MYL9, F-actin, and paxillin). This axis supports the myofibroblastic phenotype and the motile/contractile properties of CAFs. Knockdown of either YAP1 or SRC reduced cytoskeletal gene expression and α-SMA and diminished the invasion-promoting activity of CAF-conditioned media. The pro-CAF and pro-tumor roles of the YAP1–SRC–FAP/α-SMA axis were further supported in mouse co-implantation models and human-derived NF/CAF systems [113].

Second, at the maturation stage, Brunner and colleagues leveraged a patient-derived CAF biorepository to place prostate cancer CAFs along a continuum spanning an early inflammatory C1 state, an intermediate C2 state, and a late myofibroblastic C3 state. C3 myCAFs were characterized by FAP^+^, SMA^+^, and ITGA11^+^ expression, strongly activated ECM and contractile programs, and marked loss of AR, and were enriched in high-grade, highly fibrotic, and castration-resistant models. The study implicates the NF-κB–TGF-β1–YAP1 axis as a key pathway that drives progression from early CAF states toward late myCAFs and sustains their tumor-promoting phenotype. NF-κB–driven inflammatory mediators (e.g., IL6) increase TGF-β1, and TGF-β1 promotes nuclear localization and transcriptional activity of YAP1 in C2/C3, including YAP1 phosphorylation at Tyr357. In C3 myCAFs, pYAP1 appeared relatively insensitive to TGF-β receptor inhibition, consistent with a partially autonomous YAP1-activation state. This state may reinforce myofibroblastic and ECM-remodeling programs through canonical YAP target genes such as CYR61 and CTGF. Pharmacologic co-inhibition of the TGF-β receptor and YAP1 reduced expression of C3 myCAF markers (FAP, SMA, and ITGA11) and suppressed YAP target-gene programs, consistent with partial deactivation of the highly activated myCAF state. However, this perturbation also induced autophagic stress and further NF-κB activation, accompanied by increased inflammatory cytokines (e.g., IL6, IL8, and IL1B). Together, these changes suggest a positive feedback circuit linking NF-κB, TGF-β1, YAP1, autophagy, and cytokine production. Such a circuit could stabilize late myCAFs and potentially prime neighboring fibroblasts toward myCAF conversion, thereby integrating inflammatory signaling, TGF-β–driven fibrosis, and YAP1-mediated mechanotransduction [53].

Finally, at the level of functional reprogramming, Hongtao Song and colleagues divided prostate cancer CAFs into two major functional groups. ECM-CAFs were enriched for matrix-remodeling genes (e.g., CD248, COL1A1/2, CNN1, and CCN2), were driven by TGF-β signaling, and promoted extensive collagen/ECM deposition, generating a dense desmoplastic matrix that supports tumor growth. In contrast, Lym-CAFs expressed high levels of inflammatory and chemotactic factors (e.g., TNFAIP6, IL33, and CXCL8/9/10/11), were associated with lymphocyte infiltration and immune responses, and exhibited features consistent with enhanced CD8^+^ T-cell infiltration and tumor-suppressive activity. Single-cell transcriptomics and multiplex tissue staining indicated that YAP1 is highly expressed and nuclear-localized in ECM-CAFs, whereas Lym-CAFs showed NF-κB p65 nuclear translocation and upregulation of CXCL9/10/11. Pseudotime analysis further suggested a trajectory from Lym-CAFs toward ECM-CAFs, during which YAP1 expression increased in parallel with strengthening ECM programs. Mechanistically, YAP1 binds IKKα and inhibits IKKα/β phosphorylation and IκB degradation, thereby limiting NF-κB p65 activation. This repression reduces expression of Lym-CAF marker genes (e.g., CXCL9/10/11) and helps maintain the matrix-producing, tumor-promoting ECM-CAF phenotype. Upon YAP1 inhibition or deletion in ECM-CAFs, the IKK–NF-κB axis is released, leading to reduced ECM gene expression and collagen deposition. Concurrently, Lym-CAF features (e.g., TNFAIP6, IL33, and CXCL9/10/11) increase, consistent with a phenotypic shift in ECM-CAFs toward immune-activating Lym-CAFs. This transition is accompanied by increased CD8^+^ T-cell infiltration and activation, as reflected by markers such as CD25, CD69, granzyme B, and IFNγ. CD248-CreERT2 conditional Yap1 knockout mice and human-derived ECM-CAF co-implantation models provided additional support that selectively disabling YAP1 in ECM-CAFs can slow prostate tumor growth, reduce matrix stiffness, and enhance responses to anti–PD-1 therapy [111].

Collectively, YAP1 influences CAF biology across multiple stages in prostate cancer—from early lineage activation, to maintenance of late myCAFs, to reprogramming of ECM-CAFs toward immune-activating Lym-CAFs. As a regulatory hub, YAP1 may shape CAF emergence, maturation, and immunoregulatory direction, making it an attractive, stage-dependent yet broadly druggable target across CAF lineages.

### 6.2. Fibroblast Growth Factor (FGF)

The fibroblast growth factor (FGF) family comprises secreted polypeptides that signal through fibroblast growth factor receptors (FGFR1–4) to activate downstream pathways, including FRS2–RAS–MAPK and PI3K–AKT. This signaling network links cell proliferation and survival with angiogenesis, epithelial–stromal interactions, and tumorigenesis [114,115,116]. FGFs can act in paracrine, autocrine, or endocrine modes across diverse biological contexts. In prostate cancer, both tumor cells and CAFs express FGFRs and can produce FGFs. CAF-derived FGFs can activate FGFRs on tumor cells (e.g., in CWR-R1 and LNCaP) and also act autocrinely on CAFs to sustain an activated phenotype characterized by α-SMA expression and increased ECM synthesis. Together, these signals establish intertwined tumor–stroma paracrine and autocrine loops [117,118]. FGFR inhibitors can partially suppress FGFR signaling in tumor cells; however, CAFs in the surrounding stroma may continue to supply FGFs and other pro-proliferative cues. As a result, residual FGFR activity—together with compensatory signaling through parallel receptor tyrosine kinases (e.g., EGFR and PDGFR)—may preserve pro-survival programs despite FGFR blockade. Therefore, effective targeting of the FGF–FGFR axis in prostate cancer may require strategies that address CAF-driven ligand supply and pathway redundancy, rather than relying solely on FGFR inhibitors with dose-limiting toxicity [118].

In prostate cancer, FGF-related genes show widespread expression dysregulation that is associated with patient prognosis. Thus, FGF-pathway imbalance is not only a molecular feature of PCa but also carries prognostic value [119]. Baotong Zhang and colleagues delineated a mechanism of CAF reprogramming in PTEN-deficient prostate cancer centered on the FGF–FGFR1 signaling axis. The study suggested that TGF-β/SMAD–induced acetylation of KLF5 acts as a barrier to tumor progression, in part by restraining excessive FGFR1 signaling. Disruption of this acetylation via the Klf5^K358R/K369R mutations was associated with broad upregulation of FGFR1 target genes and increased levels of p-FRS2, p-ERK, and p-AKT. Single-cell and ligand–receptor analyses further indicated that FGF signaling primarily arises from paracrine secretion by inflammatory CAFs (iCAFs) toward Krt4^+^ prostate cancer cells. In the deacetylation context, FGF9 emerged as a prominently upregulated ligand. Deacetylated KLF5 increased TNF-α secretion by tumor cells, which in turn drove iCAFs toward an earlier, high-FGF9–secreting state, establishing a TNF-α–FGF9 reinforcing loop with Krt4^+^ cancer cells. Deacetylated KLF5 also upregulated CX3CR1 expression in tumor cells, further amplifying FGF9-driven FGFR1 activation. In human prostate cancer samples, high FGF9 and CX3CR1 expression was jointly associated with the FGFR1 activation marker p-FRS2. Based on these findings, the authors proposed that, in PTEN-deficient disease, targeting the FGF9–CX3CR1–FGFR1 axis (e.g., combining CX3CR1 blockade with AKT inhibition) may attenuate CAF-dependent amplification of FGF signaling and improve responses to AKT-targeted therapy [120].

Most prostate cancers are androgen receptor (AR)-dependent; however, under sustained and potent AR pathway blockade, a subset can become AR-indifferent and instead rely on alternative programs, including FGF–MAPK signaling and neuroendocrine differentiation, as exemplified by neuroendocrine prostate cancer (NEPC) and double-negative prostate cancer (DNPC; AR^−^/NE^−^) [121,122]. In a rapid-autopsy cohort, Eric G. Bluemn and colleagues classified metastatic lesions using AR and neuroendocrine markers and observed that, with widespread use of potent AR pathway inhibitors (e.g., enzalutamide and abiraterone), the proportion of double-negative metastatic prostate cancer (DNPC; AR^−^/NE^−^) increased. Transcriptomic and pathway-enrichment analyses indicated that AR target gene sets were broadly downregulated in these AR-non-dependent tumors, whereas FGF ligands and FGFR–MEK/ERK gene sets were upregulated, consistent with an alternative driver role for the FGF–FGFR–MAPK axis. Using androgen deprivation, inducible shAR, and an HSV-TK suicide system, the authors generated an LNCaP^APIPC model that lacked AR programs and did not display neuroendocrine features. In vitro, this model supported the concept that a subset of metastatic prostate cancers that have escaped AR dependence can rely on the FGF–FGFR–MAPK axis for survival, and that pathway inhibition suppresses growth of AR-indifferent tumors [123]. To incorporate microenvironmental effects, Afshan and colleagues evaluated therapeutic approaches to target FGFR signaling in the presence of cancer-associated fibroblasts (CAFs). The study used prostate cancer cell lines with distinct androgen-related features (LNCaP, VCaP, and CWR-R1) together with the immortalized CAF line PF179T to build 2D and 3D organotypic co-culture models. It then compared second-generation covalent pan-FGFR inhibitors (FIIN1/FIIN2) with a downstream FRS2α inhibitor (FRS2αi) in both tumor cells and CAFs. FGFR inhibitors inhibited proliferation and viability more strongly in tumor-cell monocultures than in CAF co-cultures. Across both 2D and 3D conditions, CAFs consistently attenuated the growth-inhibitory effects of FGFR inhibitors, with the protective effect most pronounced in LNCaP–CAF co-cultures. These results suggest that CAFs can buffer tumor cells against FGFR-targeted therapy. Although FIIN1/FIIN2 inhibited downstream FGFR signaling (p-FRS2α and p-ERK1/2) across the cell lines, 10 μM treatment was cytotoxic to both prostate cancer cells and CAFs. This pattern is consistent with nonspecific off-target effects, limiting their utility as selective tools to interrogate CAF-associated FGFR signaling. In contrast, FRS2αi (0.3–10 μM) dose-dependently reduced proliferation and metabolic activity in LNCaP–CAF co-cultures and in CWR-R1 cells, and it decreased CWR-R1 migratory capacity. In co-cultures, CAF monocultures, and 3D models, FRS2αi inhibited CAF proliferation but was substantially less cytotoxic than FIIN1/FIIN2. Notably, a fraction of CAFs survived and retained vimentin and Ki67 expression after 10 μM for 72 h or 7 μM for 6 days. Mechanistically, FRS2αi dose-dependently reduced p-FRS2α and p-ERK1/2 and increased cleaved caspase-3, consistent with blockade of activated FGFR–FRS2α signaling and induction of apoptosis. In 3D CAF co-cultures, FRS2αi reduced the area of LNCaP and CWR-R1 spheroids, decreased Ki67 expression and nuclear counts, and disrupted tumor cell–matrix interactions and overall structural integrity. Collectively, these results indicate that CAFs can attenuate the antitumor effects of FGFR inhibitors in prostate cancer. Targeting FGFR signaling downstream at FRS2α may suppress FGFR-dependent tumor cells and limit CAF-mediated pro-tumor support while sparing a subset of CAFs, providing experimental support for CAF-aware strategies to target the FGFR pathway [118].

### 6.3. NRG1

Across multiple tumor types, stroma-derived neuregulin-1 (NRG1) can activate HER3 via paracrine signaling, thereby promoting tumor-cell survival and therapy resistance under treatment pressure [124]. In prostate cancer, cancer-associated fibroblasts (CAFs) are a major component of the reactive stroma and can remodel tumor niches through paracrine networks, thereby promoting therapy resistance and malignant progression [125]. Recently, CAF-derived NRG1 has been proposed as a key microenvironmental support axis. NRG1 can bind HER3 on tumor cells and promote HER2/HER3 signaling, engaging survival pathways such as PI3K–AKT under AR inhibition and thereby sustaining survival and fostering resistance to antiandrogen therapy [126].

Under potent AR pathway inhibition (e.g., castration or second-generation antiandrogens), CAFs can provide alternative survival cues via paracrine signaling. In this context, CAF-secreted NRG1 and tumor-cell HER3 form a key pro-tumor signaling axis. Using multiple in vivo and in vitro models, Zhang and colleagues reported that CAFs promote resistance to antiandrogen therapy and identified NRG1 as a key secreted mediator acting through HER3 activation in tumor cells. Importantly, clinical-grade NRG1-neutralizing antibodies or HER3-blocking antibodies disrupted this paracrine axis in vitro and in vivo and restored sensitivity to hormone deprivation. In patients with CRPC, higher NRG1 activity was associated with poorer benefit from second-generation antiandrogens, supporting its potential utility for clinical stratification and targeted intervention [127]. Similarly, in 2024, Chunyu Wang and colleagues used patient-derived NFs/CAFs together with PC3, DU145, and enzalutamide-resistant derivatives. They found that CAF co-culture increased the enzalutamide IC50 in tumor cells and further augmented resistance in already resistant lines. Mechanistically, these effects were accompanied by higher NRG1 levels in the culture supernatant and increased HER3 phosphorylation. NRG1 knockdown in CAFs attenuated the resistance-promoting effect, whereas recombinant NRG1 rescued it, providing convergent evidence—from phenotype to pathway to causal perturbation—that CAF-derived NRG1 mediates enzalutamide resistance [128]. The study also noted higher NRG1 expression in CAFs than in NFs; however, the upstream drivers of NRG1 upregulation remain unclear and warrant further mechanistic investigation, including how CAF activation is initiated and maintained.

## 7. Biological and Clinical Significance of CAF Heterogeneity

**Figure 3:**
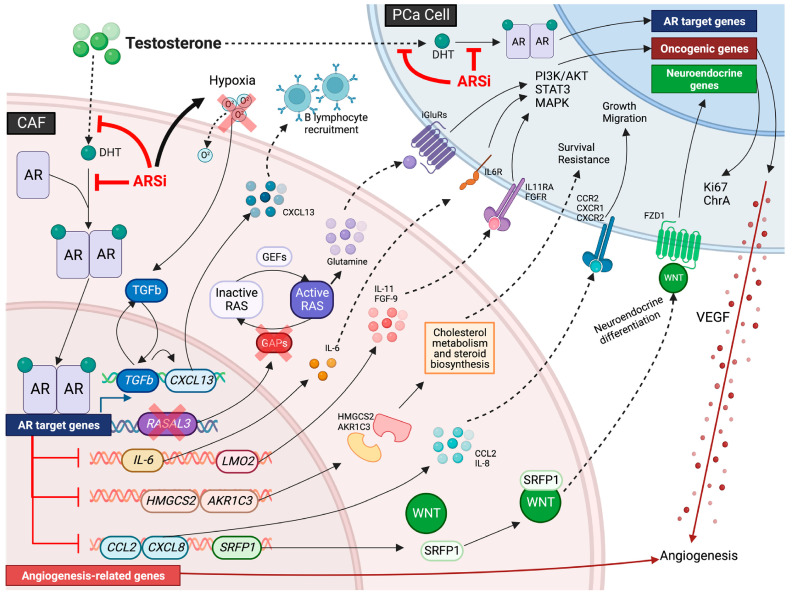
Schematic illustration of the multifaceted roles and targeting potential of CAFs in PCa. CAFs are key stromal components within the TME that actively contribute to prostate cancer progression through multiple mechanisms, including secretion of pro-tumorigenic cytokines (e.g., IL-6, CXCL8, CXCL13), remodeling of the ECM, modulation of angiogenesis-related genes, and metabolic support via cholesterol and steroid biosynthesis. CAF-derived TGF-β and chemokines activate downstream RAS and WNT signaling pathways in prostate cancer cells, enhancing oncogenic signaling (PI3K/AKT, STAT3, MAPK) and promoting cell proliferation, migration, and survival. Under androgen receptor signaling inhibition (ARSi), compensatory mechanisms such as neuroendocrine differentiation, hypoxia-induced factor activation, and immune evasion (reduced lymphocyte recruitment) are stimulated, thereby sustaining tumor growth despite androgen deprivation. CAFs further contribute to angiogenesis through upregulation of VEGF and stromal genes (HMGCS2, AKR1C3, SRFP1). Adapted from Owen, Jasmine S et al. Cancer-Associated Fibroblast Heterogeneity, Activation and Function: Implications for Prostate Cancer. Biomolecules. Copyright © 2024 by the authors [133]. This article is an open access article distributed under the terms and conditions of the Creative Commons Attribution (CC BY) license.

CAFs play crucial roles in the initiation and progression of PCa. By secreting protumorigenic factors, remodeling the ECM, providing metabolic and exosome-mediated support, and shaping an immunosuppressive microenvironment, they collectively drive cancer progression [129]. At present, selective markers for targeting CAFs are lacking. Moreover, CAFs are not uniformly tumor-promoting—some subpopulations restrain tumors—so indiscriminate or prolonged CAFs suppression may paradoxically accelerate tumor progression [130]. Nevertheless, as central organizers within the TME, CAFs remain attractive therapeutic nodes to modulate immune pathways, potentiate immunotherapy, and dismantle stromal barriers [131,132] (Figure 3).

### 7.1. CAF-Based Prognosis and Immunotherapy Prediction

Accumulating evidence suggests that cancer-associated fibroblasts (CAFs) are not passive background stroma in prostate cancer but actively shape prognosis and responses to immunotherapy. Therapeutic modalities—including androgen deprivation, AR inhibition, chemotherapy, and radiotherapy—can themselves reprogram CAFs and activate convergent resistance-associated pathways. Accordingly, integrating CAF phenotypes and their dominant signaling programs into stratification frameworks may provide a biological basis for prognostic assessment and immunotherapy prediction, help identify high-risk patients, and guide optimization of immunotherapy and combination regimens in prostate cancer [133].

Pan-cancer single-cell atlas studies suggest that CAFs can be grouped into six functional subtypes with relatively conserved transcriptional programs (CAFmyo, CAFinfla, CAFadi, CAFEndMT, CAFPN, and CAFap). These subtypes can also be organized along the activation continuum from normal fibroblasts (NFs) to CAFs into three transitional states (CAFstate1–3), providing a shared framework for comparing CAF heterogeneity across cancer types and data platforms [134]. Using this taxonomy, Ze Gao and colleagues derived marker-gene sets from scRNA-seq and computed six subtype-specific CAF scores (CAFmyo, CAFinfla, CAFadi, CAFEndMT, CAFPN, and CAFap) in multiple bulk RNA-seq cohorts. In TCGA-PRAD, higher scores for each subtype were associated with shorter progression-free interval (PFI), and these associations were validated in independent cohorts (e.g., MSKCC, CPGEA, and GSE70770). The six subtype scores were further aggregated into a total CAF score (tCAF), stratifying patients into tCAF-high and tCAF-low groups. Across four prostate cancer cohorts, the tCAF-high group had poorer PFI, showed enrichment of cell proliferation–related pathways, and was associated with adverse clinicopathologic features (higher Gleason score and more advanced T and N stages). Collectively, these findings suggest that higher CAF burden tracks with more aggressive disease biology. Immunologically, immune status was evaluated using ssGSEA, ESTIMATE, the immune phenotype score (IPS), and cytolytic activity (CYT) metrics. Tumors with high tCAF showed greater enrichment of immune-related gene sets and higher stromal, immune, and ESTIMATE scores, as well as elevated CYT. Together, these results are consistent with an “immune-infiltrated but constrained” microenvironment. IPS analyses suggested that, in the context of CTLA4 expression, the tCAF-low group was predicted to respond better to immunotherapy, whereas differences between tCAF groups were not evident when stratified by PD-L1 expression. At the gene level, CTLA4 expression was higher in tCAF-high tumors, whereas NECTIN2 was relatively higher in the tCAF-low group. In contrast, PD-L1 and PD-L2 differed minimally between groups. Most major MHC genes were more highly expressed in tCAF-high tumors, consistent with increased antigen-presentation capacity [135]. Wenhao Wang and colleagues integrated prostate cancer single-cell and bulk RNA-seq data and focused on antigen processing/presentation genes. They identified a CAF subtype with antigen processing and presentation features—CTSK^+^MRC2^+^ CAF-C1 (APPCAF)—and contrasted it with a non-antigen-presenting CAF cluster (NonAPP-CAF-C2) to derive 55 APPCAF-related genes (APPCAFRGs). Using univariate Cox regression, the authors selected 20 APPCAFRGs associated with biochemical recurrence (BCR) and performed NMF clustering in TCGA-PRAD. This APPCAF-informed subtyping separated patient groups with distinct BCR outcomes. The resulting risk score was validated in multiple cohorts (TCGA-PRAD, GSE116918, and GSE70769), where it predicted BCR and remained an independent prognostic factor in multivariable Cox models. This APPCAF-centered CAF prognostic signature may help identify patients at high risk of biochemical recurrence, and its interplay with the immune microenvironment may also contribute to progression toward castration resistance [58]. Together, these studies illustrate two complementary stratification layers. The six-subtype–based tCAF score captures the overall CAF burden and its associations with prognosis and immune constraint, whereas the CTSK^+^MRC2^+^ APPCAF–anchored APPCAFRS refines risk assessment by focusing on antigen presentation and immune regulation. This functional lens may help explain CAF-associated variation in biochemical recurrence risk and predicted immunotherapy responsiveness.

### 7.2. Targeting CAFs Paracrine Signaling and Soluble Support

In PCa, CAFs secrete diverse soluble cues that profoundly influence tumor initiation, progression, and metastasis. These effects include promoting tumor-cell proliferation and invasion, inducing angiogenesis, facilitating metastasis and therapeutic resistance, and supporting metabolic reprogramming [136,137,138]. CAFs are major sources of paracrine mediators—e.g., TGF-β, CXCL12, IL-6, VEGF—that act as shared upstream drivers of multiple survival and resistance pathways [35,131,139]. Therefore, targeting CAFs can concurrently disrupt multiple downstream driver cascades, rather than merely inhibiting a single mutant pathway or receptor.

TGF-β–activated CAFs shape an immune-excluded TME and are key drivers of resistance to ICB. Preclinical models show that blockade of PD-L1 or TGF-β alone has limited efficacy, whereas their combined inhibition increases intratumoral T cell infiltration and reduces tumor burden [140]. In 2025, Qiu and colleagues used scRNA-seq to profile CAFs in CRPC and found pronounced upregulation of TGF-β signaling in CRPC-CAFs. Relative to PCa-CAFs, CRPC-CAFs showed functional shifts, including enhanced extracellular-matrix remodeling and collagen biosynthesis. Gene-set enrichment analysis (GSEA) indicated strong activation of TGF-β signaling in CRPC-CAFs, driving CAF proliferation and collagen remodeling and reinforcing the immunosuppressive milieu, thereby diminishing responses to immunotherapy. Targeting the TGF-β pathway—particularly within CAFs—emerges as a strategy to improve immunotherapy efficacy. In mouse models, anti–TGF-β monotherapy inhibited tumor growth and increased immune-cell infiltration. When combined with anti–PD-1 ICB, anti–TGF-β therapy further augmented antitumor immunity [24]. Together, these findings indicate that targeting TGF-β signaling in CAFs can improve the TME and enhance immunotherapy, representing an emerging strategy for CRPC.

Across multiple cancers, the CXCL12–CXCR4 axis is implicated in tumor-cell survival, proliferation, migration, angiogenesis, and metastasis. In CAFs, autocrine CXCL12 signaling helps maintain an α-SMA^+^ myofibroblastic phenotype and a high-output secretory program, positioning this pathway as a key hub that supports malignant dissemination [141,142]. Building on this rationale, Jiayan Lang and colleagues developed a self-assembling nanoparticle (PNP/siCXCL12/mAb) featuring a cholesterol-modified nona-arginine cell-penetrating peptide core and a surface-conjugated anti–FAP-α monoclonal antibody to enable selective delivery of siCXCL12 to FAP^+^ prostate CAFs. In CAFs, CXCL12 mRNA and secreted protein levels decreased by ~60–70%, accompanied by reduced α-SMA expression and G0/G1 cell-cycle arrest, consistent with a shift toward a less activated CAF state. Protein-array profiling indicated that CXCL12 silencing broadly reduced pro-tumor mediators in CAF-conditioned medium (e.g., IL-6, TGF-β1, VEGF, FGF, EGF, and MCP-1) while increasing anti-angiogenic factors such as serpin B5 and thrombospondin-2. Functionally, conditioned medium from CXCL12-silenced CAFs reduced PC-3 wound closure and Matrigel invasion, as well as HUVEC migration and tube formation; several effects were partially rescued by exogenous CXCL12. In an orthotopic PC-3Luc+CAF prostate model, intravenous PNP/siCXCL12/mAb reduced stromal CXCL12 expression and the α-SMA^+^ CAF area without overt systemic toxicity. Treatment also decreased CD31^+^ neovessel density, inhibited primary tumor growth, and reduced metastatic lesions in distant organs (e.g., liver, kidney, and intestine). The authors proposed that, instead of broadly depleting CAFs, targeting the CAF-derived CXCL12 axis could functionally normalize CAFs and reprogram their secretome. Such an approach may concurrently weaken tumor invasion, metastasis, and angiogenesis through multiple downstream pathways, supporting CAF-focused microenvironment remodeling as a strategy to restrain prostate cancer progression [143]. Similarly, using prostate cancer as a model, Juanjuan Li and colleagues showed that interventions targeting the CAF-associated CXCL12–CXCR4 pathway can remodel both the stromal and immune microenvironment. They developed a degradable, redox-sensitive diselenide-bonded organosilicon nanosystem (Se@A&F) that encapsulated the CXCR4 antagonist AMD3100 and used surface-conjugated anti–FAP-α antibody for CAF-targeted delivery. This system reduced CXCR4 expression in CAFs and triggered downstream changes consistent with CAF deactivation, including attenuation of the dense ECM barrier and the immunosuppressive cytokine milieu. These changes shifted the microenvironment from a pro-tumor, immune-excluded state toward a more permissive context for immune attack. Functionally, blockade of the CAF-associated CXCL12–CXCR4 pathway inhibited tumor-cell migration, invasion, and angiogenesis, accompanied by slower orthotopic tumor growth and reduced metastasis. It also increased intratumoral immune infiltration—particularly T cells—and enhanced antitumor activity, with proposed mechanisms including improved antigen availability, activation of cytotoxic T lymphocytes, and reprogramming of tumor-associated macrophages toward an M1-like phenotype. Collectively, this work provides preclinical support for a multi-pronged strategy in which CAF normalization via disruption of the CXCL12–CXCR4 pathway enhances immune infiltration while constraining tumor growth and metastasis [144].

VEGF-A (encoded by VEGFA) not only drives angiogenesis but also contributes to immune evasion and metastasis, making it a key target in cancer research [145]. Therapeutic strategies targeting VEGF-A or its receptors have become established approaches in cancer therapy. In 2022, Ma and colleagues reported that ligustilide—the major active constituent of the volatile oils of Angelica sinensis (Danggui) and Ligusticum chuanxiong (Chuanxiong)—modulates TLR4 signaling in PCa-CAFs, attenuating the downstream p38/JNK/ERK–AP-1 cascade and downregulating VEGF-A expression in CAFs. This change does not alter CAFs proliferation per se but weakens their pro-proliferative, promigratory, and tube-forming effects on HUVECs, resulting in reduced tumor vascular density in vivo. Mechanistically, ligustilide decreases glycolytic activity and HIF-1α levels in CAFs, further suppressing VEGF-A secretion. These findings support a novel anti-angiogenic strategy: precise “source reduction” of VEGF-A production within the TME—rather than antibody neutralization—using a natural bioactive compound [137].

In prostate epithelial cells with dual loss of PTEN and TP53, Yanushko and colleagues observed that tumor-associated CAFs provide crucial paracrine support during tumor progression. Single-cell transcriptomics with ligand–receptor inference showed that iCAFs markedly upregulate IL-6, whereas the epithelial plasticity subset (EMTc) highly expresses the IL-6 receptor (IL6R), establishing an iCAF(IL-6)→EMTc(IL-6R) signaling axis. Functionally, conditioned medium from double-knockout (DKO)–derived CAFs induced a partial EMT (vimentin-positive) phenotype in DKO epithelial organoids; this effect was reversed by the JAK inhibitor ruxolitinib or by IL-6–neutralizing antibodies. Consistently, enzyme-linked immunosorbent assay (ELISA) showed that CAFs secreted higher IL-6 levels than epithelial organoids, with DKO-derived CAFs producing even more. In addition, exogenous IL-6 induced vimentin expression only in DKO organoids, not in organoids with PTEN loss alone. Patient single-cell data and assays in patient-derived DKO organoids concordantly supported a model in which CAF-secreted IL-6 promotes epithelial plasticity by activating JAK/STAT3 signaling. Collectively, these findings indicate that, in this genetic context, CAF-derived IL-6 operates as a key cytokine pathway driving epithelial plasticity and invasiveness, suggesting that targeting CAFs to block the IL-6/JAK/STAT3 axis could diminish soluble-factor support to tumor cells and impede progression [146]. In 2025, Gao and colleagues reported that CAFs promote PCa cell migration and invasion by secreting the inflammatory cytokine IL-17A. Compared with NFs, CAFs secrete higher levels of IL-17A, thereby activating the SMAD3/p38 MAPK signaling pathway, inducing EMT, enhancing cancer-cell migration and invasion, and promoting tumor growth and lung metastasis in vivo. This work reveals a CAF–IL-17A–SMAD3/p38 MAPK-dependent mechanism and identifies a potential therapeutic target in PCa [147].

Collectively, these studies converge on a consistent framework. First, they indicate that prostate cancer-associated fibroblasts (CAFs) can function as upstream hubs that secrete paracrine mediators—including TGF-β, the CXCL12–CXCR4 axis, VEGF-A, IL-6, and IL-17A—that promote extracellular matrix remodeling, angiogenesis, epithelial plasticity, metastasis, and immune exclusion. Second, they suggest that selectively targeting these pathways at their stromal source—using antibodies, small-molecule inhibitors, or fibroblast activation protein (FAP)-guided nanosystems—can normalize CAF function and remodel the tumor microenvironment (TME). This remodeling is characterized by reduced fibrosis and improved drug penetrance, suppression of pro-angiogenic and pro-invasive signaling, and enhanced infiltration and activation of cytotoxic immune cells. In this view, paracrine reprogramming of CAFs extends beyond blocking individual cytokines. Instead, it represents a strategy to concurrently dismantle multiple survival and resistance programs, thereby creating a microenvironment that is more amenable to therapy in advanced and castration-resistant prostate cancer.

### 7.3. Targeting CAF-Driven Stromal Barriers

In the TME, CAFs continuously secrete ECM components—such as collagens and fibronectin—driven by pathways including TGF-β, resulting in excessive ECM deposition with impaired degradation and formation of a dense fibrotic stroma [148]. This dense, highly crosslinked ECM establishes a physical barrier that limits diffusion and penetration of therapeutics (e.g., chemotherapeutics, nanomedicines) and impedes effector immune-cell infiltration (e.g., CD8^+^ T cells) into the tumor core, thereby weakening antitumor immunity [149]. In addition, oxidative stress and metabolic reprogramming further stimulate CAFs to secrete ECM, exacerbating fibrosis. As ECM accumulates, stromal stiffness and solid stress increase, creating conditions conducive to invasion and metastasis and reinforcing malignant phenotypes. CAFs also enhance local immune evasion by secreting immunosuppressive mediators (e.g., CXCL12, IL-6), which in turn promotes further ECM deposition [150]. Therefore, dismantling CAF-mediated physical barriers is a promising strategy to enhance penetration of chemotherapy, targeted agents, and immunotherapies.

S100A11, a member of the S100 calcium-binding protein family, contains EF-hand helix–loop–helix motifs and typically dimerizes and oligomerizes. S100A11 is associated with tumor-cell proliferation, migration, invasion, and therapeutic resistance across multiple cancers [151]. In 2024, Dali Han and colleagues investigated S100A11. They found that CAFs are abundant in PCa tissues, where collagen deposition accompanies formation of an immune-excluded microenvironment. S100A11 is highly expressed in both tumor cells and CAFs. Its knockdown reduces α-SMA^+^ CAFs, weakens the stromal barrier, and increases intratumoral T cell infiltration—particularly GZMB^+^ and IFN-γ^+^ effector CD8^+^ T cells. Knocking down S100A11 only in tumor cells decreased a subset of CAFs and increased CD4^+^ T cells, but improvements in tumor volume and CD8^+^ infiltration were limited. By contrast, simultaneous knockdown in both tumor cells and CAFs more substantially reduced CAFs and markedly enhanced CD4^+^/CD8^+^ infiltration, accompanied by tumor shrinkage. Mechanistically, S100A11 knockdown did not directly suppress FGFR–PI3K–AKT signaling but sensitized CAFs to the FGFR inhibitor erdafitinib, as evidenced by reduced PI3K/AKT pathway activity, impaired migration and invasion, and increased apoptosis. Combination therapy with erdafitinib further reduced CAFs, lowered PD-L1 expression, and amplified effector CD8^+^ T cell responses, shifting the PCa microenvironment from immune exclusion to an inflamed state and yielding a stronger antitumor effect [152].

### 7.4. Targeting CAF-Derived Exosomes

CAF-derived exosomes carry specific proteins and non-coding RNAs that activate signaling pathways in recipient cells and influence cancer progression. During tumor evolution, they can promote or inhibit tumor growth and metastasis (e.g., miR-210, miR-93-5p, miR-181d-5p), confer chemoresistance by transferring resistance-related molecules (e.g., miR-106b, miR-21, lncRNA H19, UCA1), and mediate immune evasion via PD-L1 and miR-92–driven suppression of T cells [153]. Compared with directly targeting tumor cells, intervening in CAF-derived exosomes offers several advantages. First, because CAF exosomes act as shared upstream drivers, blocking their signals can simultaneously suppress multiple oncogenic pathways and yield broad-spectrum antitumor effects. In addition, specific markers on CAF exosomes (e.g., FAP, PDGFRβ) provide handles for precise delivery of inhibitors or RNA-interference (RNAi) cargo, increasing therapeutic selectivity and limiting off-target injury to normal tissues. Therefore, targeting CAF-derived exosomes may inhibit PCa growth and metastasis and enhance the efficacy of radiochemotherapy and immunotherapy, offering substantial potential for clinical translation [154].

Studies of exosomes show that CAF-derived exosomes promote PCa progression by delivering protumor miRNAs and depleting tumor-suppressive miRNAs. For example, miR-432-5p promotes chemoresistance by suppressing ferroptosis via targeting CHAC1. miR-432-5p is enriched in CAF-derived exosomes and modulates ferroptosis by regulating CHAC1 expression [155]. Ferroptosis—an iron-dependent cell death marked by lipid peroxidation—underlies the activity of multiple anticancer therapies [156]. Thus, antagonizing miR-432-5p represents a potential therapeutic approach in PCa. Suppressing miR-432-5p may restore chemosensitivity and improve treatment outcomes. Mechanisms driving post-ADT castration resistance and metastasis are of high clinical relevance. In 2020, Zhang and colleagues reported that ADT reduces miR-146a-5p in exosomes secreted by prostate CAFs. Under basal conditions, this microRNA targets EGFR, restraining EGFR/ERK signaling and inhibiting cancer-cell EMT, migration, and invasion. Loss of exosomal miR-146a-5p after ADT derepresses EGFR/ERK, leading to sustained activation, upregulation of EMT markers (vimentin, N-cadherin, MMP-2/9), and downregulation of E-cadherin, thereby enhancing metastatic potential. In vitro and in vivo, supplementation or overexpression of miR-146a-5p reversed this prometastatic effect. These findings implicate the exosomal miR-146a-5p–EGFR–ERK axis in post-ADT progression and suggest a combination strategy: augmenting CAF-derived exosomal miR-146a-5p alongside ADT to suppress metastasis and improve therapeutic efficacy [157].

Ligand-directed tumor-suppressive miRNA replacement as a complementary strategy. Beyond modulating CAF-derived exosomal signals, restoring tumor-suppressive miRNAs in prostate cancer cells represents a mechanistically complementary intervention when the microenvironment drives loss of suppressive miRNA activity. Abdelaal et al. developed a vehicle-free, PSMA-targeted delivery platform by conjugating miR-34a to the high-affinity small-molecule ligand DUPA and integrating the ionophore nigericin to promote endosomal escape. While DUPA–miR-34a achieved PSMA-dependent uptake and target repression, incorporation of nigericin increased cytosolic miR-34a enrichment and accelerated functional silencing, resulting in measurable antiproliferative activity in vitro and delayed tumor growth in LNCaP xenografts upon systemic administration. Importantly, a fully chemically stabilized miR-34a (2′-O-methyl/2′-fluoro and phosphorothioate pattern) further enhanced in vivo efficacy, highlighting that successful miRNA replacement requires coordinated solutions for tumor targeting, endosomal release, and nuclease stability [158].

## 8. Discussion

PCa is the fifth leading cause of cancer death in men worldwide. In this review, we delineate the multifaceted roles of CAFs in PCa, including immune modulation, ECM remodeling, and promotion of angiogenesis. CAFs are pivotal in PCa initiation and progression and also hold substantial promise for prognostic assessment and future therapeutic strategies. CAFs lineages and subtypes (e.g., iCAFs, myCAFs) differ in immune regulation, ECM remodeling, and metabolic support; across tumor models, indiscriminate ablation of a single CAFs subtype can paradoxically accelerate tumor progression [60,159]. CAFs also couple tumor-cell plasticity to immune suppression through mediators such as IL-6/JAK–STAT3, TGF-β, and CXCL12; after blockade of a single node, alternative routes are often activated, leading to compensatory escape [9]. The CAF-dominated dense ECM limits drug penetration, whereas systemic inhibition of the CAF–ECM axis risks disrupting ECM-dependent tissue homeostasis and structural integrity [160]. Accordingly, strategies should shift from wholesale depletion to function- and subtype-directed precision interventions, combined with multipathway co-blockade and companion diagnostics, to maximize clinical benefit while preserving physiological homeostasis.

At present, CAFs exhibit pronounced heterogeneity and plasticity, and the field lacks robust markers and stratification; the evidence base remains limited, making single-target depletion of specific CAFs subtypes unrealistic. Moreover, the double-edged-sword effect of CAFs depletion remains unresolved [161,162,163]. Future strategies for targeting CAFs will require breakthroughs that balance precise identification, functional intervention, and safety. On one hand, single-cell multi-omics, spatial transcriptomics, and in vivo imaging can build dynamic atlases of CAFs states and subtypes, enabling targeted identification of protumor phenotypes [41,134,164]. On the other hand, strategies should shift from indiscriminate depletion to reversible functional reprogramming—using small molecules, nucleic acids, or metabolic modulation—to convert protumor CAFs into homeostatic or tumor-restraining phenotypes [165,166]. In parallel, programmable nanocarriers and degradable local-delivery platforms can co-modulate signaling axes and ECM mechanics within the tumor niche, thereby reducing barriers to drug penetration and immune suppression while preserving stromal homeostasis in normal tissues [167]. Ultimately, a composite strategy—subtype stratification + functional reprogramming + localized precision delivery—could maximize efficacy, minimize adverse effects, and delay the emergence of therapeutic resistance.

## 9. Conclusions

Prostate cancer progression, particularly to castration-resistant disease, is tightly coupled to tumor-microenvironment remodeling in which cancer-associated fibroblasts (CAFs) shape immune suppression, extracellular-matrix dynamics, angiogenesis, and treatment resistance. Single-cell and spatial multi-omics highlight marked CAF heterogeneity and plasticity, supporting a shift from indiscriminate stromal depletion to subtype- and function-guided targeting, barrier modulation, and reversible CAF reprogramming. Future efforts should prioritize biomarker-driven patient stratification and rational combination strategies that integrate CAF-directed interventions with systemic therapies while preserving stromal homeostasis.

## Figures and Tables

**Table 1:** Overview of Key Information on Cancer-Associated Fibroblasts in Prostate Cancer.

CAF Subtype	Origins/Sources	Key Markers/ Expression Features	Primary Functions/ Mechanisms	Specific Roles in PCa (Ultimate Outcomes)	Associated Signaling Pathways	Therapeutic Potential/ Targets
**Mainly Studied Subtypes**						
MyCAFs	Normal fibroblasts or mesenchymal stem cells induced by tumor epithelial factors (e.g., TGF-β, FGF); also via EMT or endothelial-mesenchymal transition (EndMT) [64,65,66].	α-SMA, TAGLN, MYL9, TPM1, COL1A1, COL1A2, MAOA ↑; low AR expression ↓ in some contexts [67].	ECM remodeling, immune suppression, proliferation/migration [67].	Accelerates castration-resistant prostate cancer (CRPC) recurrence, immune escape, and tumor invasion [10].	TGF-β/HIF-1α/CTGF ↑ (positive feedback) [55]; MAOA-ROS-WNT5A ↑ (immunosuppressive phenotype) [68]; NF-κB-TGFβ1-YAP1 ↑ (AR downregulation ↓) [53].	Inhibit MAOA to enhance T cell activation [68]; block TGF-β to prevent activation [55]; combine with YAP1 inhibitors to restore enzalutamide sensitivity [53]; target CTHRC1 for ADT resistance [54].
iCAFs	Local normal fibroblasts, mesenchymal stem cells (MSCs), endothelial cells (via EndMT), adipocytes, or bone marrow precursors; induced by IL-1, TGF-β1, CXCL16, lactate, or oxidative stress (ROS) [13,51,69].	PDGFRA, LIF, CXCL1 ↑, CXCL12 ↑, CXCL14 ↑, CCL2 ↑, IL-6 ↑, IL-8 ↑, IL-1β ↑, CSF2, CSF3 [13]; HSD17B2 ↑ in CRPC [51].	Inflammation, immune cell recruitment, angiogenesis [13,51,69]	Promotes CRPC progression [51], ADT resistance, and tumor invasion [8,69,70].	IL-1/NF-κB ↑ [71]; TGF-β/SMAD/SOX4 ↑ (phenotype conversion) [8]; JAK1/STAT3 ↑ (inflammation regulation) [70]; AR-TGFBR1 ↓ (ADT-induced TGFBR1 ↑ sensitivity) [8].	Target HSD17B2 to reduce AR inhibition [51]; inhibit TGF-β/SPP1/ERK to block resistance [8]; upregulate HHIP to suppress JAK1/STAT3 ↑ [70]; knock down ELF3 to reduce proliferation [69].
apCAFs	Primarily from mesothelial cells via IL-1/NF-κB and TGF-β/Smad pathways; through mesothelial-to-mesenchymal transition (MMT) [72].	MHC-II ↑, CD74 ↑ [73]; co-expression of mesothelial and CAFs markers (e.g., CTSK, MRC2) [58].	Antigen presentation, T cell modulation, immune evasion [58].	Enhances immune evasion, reducing anti-tumor immunity post-ADT [58].	MHC-II pathway ↑ (pre-ADT); ligand-receptor interactions with CD4^+^ T cells ↑ (TCR activation via CD25/CD69 ↑) [58].	Restore apCAF function post-ADT to enhance CD4^+^ T cell activation; combine with ICB; use as prognostic marker [58].
meCAFs	Local fibroblasts influenced by oncogenic signals (RAS, TGF-β, NF-κB) via oxidative stress; hypoxia-induced epigenetic changes (HIF-1α-dependent) [74,75,76].	MCT4 ↑ (lactate export) [77]; ACLY ↑, ACC ↑, FASN ↑ (lipid synthesis) [78]; SIRT3 ↓ [77]; glutamine/aspartate synthesis enzymes (ASNS ↑, PC ↑) [79,80].	Metabolic reprogramming [77], amino acid/lipid support [78,79,80].	Fuels tumor growth [80], EMT [81], ADT resistance [79], and metastasis [81].	HIF-1α/ROS ↑ (lactate production) [77]; SIRT1/PGC-1α ↑ (mitochondrial biogenesis) [81]; mTOR-FOXM1 ↑ (glutamine-induced NE shift) [79]; ATF4 ↑ (aspartate/asparagine synthesis) [80].	Target MCT1/ACLY to disrupt lactate-lipid axis [78]; inhibit glutamine synthesis via RASAL3 [79]; block mitochondrial transfer to reduce OXPHOS [81].
**Other Minor Subtypes**						
FerroCAFs [82]	These subtypes are primarily identified through single-cell RNA sequencing, with limited detailed studies on their origins.	PVR (CD155) ↑, Hmox1 ↑; high Fe^2+^ content.	Iron metabolism, immunosuppression	Promotes immunosuppression and poor prognosis in metastatic CRPC.	Hmox1-Kdm6b ↑ (epigenetic MASP upregulation).	Inhibit Hmox1/Kdm6b to reduce immunosuppression and enhance ICB efficacy.
mCAFs [83]		Low α-SMA ↓; high ECM/EMT-related genes ↑ (specific markers not fully listed).	ECM remodeling, immunosuppression, NE differentiation	Drives Enzalutamide resistance and neuroendocrine differentiation in CRPC.	Immune/NE pathways ↑ (specific pathways not detailed).	Target immune/NE pathways to reduce resistance; use as CRPC biomarker.
C0 CAFs [62]		aSMA ↑, CAV1 ↑, MYH1 ↑, MCAM ↑, RGS5 ↑.	Microvascular development, ECM remodeling.	Supports microvascular development in HSPC, contributing to tumor growth.	Microvascular-related pathways ↑ (specific pathways not detailed).	Target microvascular pathways to inhibit tumor growth; use as HSPC biomarker.
C1 CAFs [62]		COL1A1 ↑, COL3A1 ↑, FN1 ↑, FAP ↑.	ECM remodeling, tumor invasion	Drives CRPC progression and poor prognosis via enhanced ECM remodeling.	ECM remodeling pathways ↑ (specific pathways not detailed).	Target FN1/FAP to reduce ECM remodeling and CRPC progression; use as prognostic marker.
ABCA8+ CAFs (C1-like CAFs) [84]		ABCA8 ↑, NFAT5 ↑.	Proliferation, migration	Enhances tumor growth and metastasis via proliferation and invasion.	PTN-NCL-NFAT5 ↑ (CAFs conversion and progression).	Target NFAT5 to suppress proliferation/invasion; use as CRPC biomarker.

Note: ↑ and ↓ indicate upregulation (increased expression/activity) and downregulation (decreased expression/activity), respectively, as reported in the cited studies (relative to the corresponding controls/baseline).

## Data Availability

No new data were created or analyzed in this study. Data sharing is not applicable to this article.

## Abbreviations

The following abbreviations are used in this manuscript:

ADT	Androgen deprivation therapy
AR	Androgen receptor
CAF(s)	Cancer-associated fibroblast(s)
CRPC	Castration-resistant prostate cancer
ECM	Extracellular matrix
EMT	Epithelial–mesenchymal transition
EndMT	Endothelial–mesenchymal transition
FN1	Fibronectin 1
HSPC	Hormone-sensitive prostate cancer
ICB	Immune checkpoint blockade
iCAF(s)	Inflammatory cancer-associated fibroblast(s)
MAOA	Monoamine oxidase A
meCAF(s)	Metabolic cancer-associated fibroblast(s)
MRI	Magnetic resonance imaging
mCRPC	Metastatic castration-resistant prostate cancer
myCAF(s)	Myofibroblastic cancer-associated fibroblast(s)
NE	Neuroendocrine
NF(s)	Normal fibroblast(s)
OXPHOS	Oxidative phosphorylation
PCa	Prostate cancer
PSA	Prostate-specific antigen
TAM(s)	Tumor-associated macrophage(s)
TAN(s)	Tumor-associated neutrophil(s)
TCA	Tricarboxylic acid
TGF-β	Transforming growth factor beta
TME	Tumor microenvironment
Treg(s)	Regulatory T cell(s)
vCAF(s)	Vascular cancer-associated fibroblast(s)
